# Diversification and Species Boundaries of *Rhinebothrium* (Cestoda; Rhinebothriidea) in South American Freshwater Stingrays (Batoidea; Potamotrygonidae)

**DOI:** 10.1371/journal.pone.0022604

**Published:** 2011-08-03

**Authors:** Florian B. Reyda, Fernando P. L. Marques

**Affiliations:** 1 Department of Ecology and Evolutionary Biology, University of Connecticut, Storrs, Conneticut, United States of America; 2 Laboratório de Helmintologia Evolutiva, Departamento de Zoologia, Instituto de Biociências, Universidade de São Paulo, São Paulo, Brazil; 3 Biology Department and Biological Field Station, State University of New York College at Oneonta, Oneonta, New York, United States of America; Ecole Normale Supérieure de Lyon, France

## Abstract

**Background:**

Neotropical freshwater stingrays (Batoidea: Potamotrygonidae) host a diverse parasite fauna, including cestodes. Both cestodes and their stingray hosts are marine-derived, but the taxonomy of this host/parasite system is poorly understood.

**Methodology:**

Morphological and molecular (Cytochrome oxidase I) data were used to investigate diversity in freshwater lineages of the cestode genus *Rhinebothrium* Linton, 1890. Results were based on a phylogenetic hypothesis for 74 COI sequences and morphological analysis of over 400 specimens. Cestodes studied were obtained from 888 individual potamotrygonids, representing 14 recognized and 18 potentially undescribed species from most river systems of South America.

**Results:**

Morphological species boundaries were based mainly on microthrix characters observed with scanning electron microscopy, and were supported by COI data. Four species were recognized, including two redescribed (*Rhinebothrium copianullum* and *R. paratrygoni*), and two newly described (*R. brooksi* n. sp. and *R. fulbrighti* n. sp.). *Rhinebothrium paranaensis* Menoret & Ivanov, 2009 is considered a junior synonym of *R. paratrygoni* because the morphological features of the two species overlap substantially. The diagnosis of *Rhinebothrium* Linton, 1890 is emended to accommodate the presence of marginal longitudinal septa observed in *R. copianullum* and *R. brooksi* n. sp. Patterns of host specificity and distribution ranged from use of few host species in few river basins, to use of as many as eight host species in multiple river basins.

**Significance:**

The level of intra-specific morphological variation observed in features such as total length and number of proglottids is unparalleled among other elasmobranch cestodes. This is attributed to the large representation of host and biogeographical samples. It is unclear whether the intra-specific morphological variation observed is unique to this freshwater system. Nonetheless, caution is urged when using morphological discontinuities to delimit elasmobranch cestode species because the amount of variation encountered is highly dependent on sample size and/or biogeographical representation.

## Introduction

### The context

The central unit for taxonomy and systematics is the species, and assigning populations unequivocally to species is essential for a meaningful reference system of biological information [1], [2]. Consequently, methods to objectively and rigorously delimit species in nature are required for reliable species circumscriptions [3]. Recognition of species boundaries is important to areas outside of taxonomy because species are frequently used as fundamental units of analysis in biogeography, ecology, macroevolution and conservation biology [4]–[12]. For example, coevolutionary studies in which historical patterns of host and parasite association are inferred based on host specificity, or other parameters, are completely dependent on correct host and parasite species identifications [13]. Although species criteria, definitions, and delineations have been contentiously debated for decades ([14], [15] among many others), the recognition of species boundaries is primarily influenced by the method used to delimit species [16]. Integrative and pluralistic approaches to species delineation in which data are acquired and synthesized from different and independent sources in conjunction with appropriate methods of extracting information from the data gathered (see [2], [17]) have the potential to enhance species discovery and our understanding of biological diversity.

### The problem

Neotropical freshwater stingrays (Batoidea: Potamotrygonidae) are the only family of elasmobranchs entirely restricted to freshwater [18]. Potamotrygonids include 4 genera consisting of over 20 species [19]–[26], and occur within nearly all of the major river systems that drain into the Atlantic Ocean or Caribbean Sea [18], [19]. Potamotrygonids, like their marine batoid counterparts, are host to a diversity of metazoan parasites (reviewed in [27]), the most diverse of which are the cestodes, which include 20+ species [27], [28]. The cestode species that parasitize potamotrygonids include a trypanorhynch [29], as well as tetraphyllideans (considered paraphyletic, see [30]–[34]), and nine species representing two genera, *Rhinebothrium* Linton, 1890 and *Rhinebothroides* Mayes, Brooks and Thorson, 1981, of the newly erected order Rhinebothriidea Healy, Caira, Jensen, Webster and Littlewood, 2009. Each of these cestode groups is restricted to elasmobranch hosts. The cestode species that parasitize potamotrygonids are therefore more closely related to cestodes of marine elasmobranchs than they are to cestodes species that parasitize other river-dwelling hosts, such as teleosts (e.g., protoecephalidean cestodes of catfishes), with which they co-occur in the rivers, presumably during the egg stage of their life cycles.

Although extant potamotrygonids and their cestodes are generally believed to be the descendants of marine ancestors, the history of colonization remains widely contested. This issue has been one of the primary foci of investigations in this host/parasite system, which included the use of phylogenies of marine and freshwater cestodes to infer host phylogenies [35] as a method towards uncovering patterns of colonization. Such studies were limited, however, by the preliminary nature of the taxonomy of both stingrays and their parasites. At the time of the analysis by Brooks et al [35] many potamotrygonid species were inadequately characterized or taxonomically confused. Subsequent work [18]–[26] has resulted in improved potamotrygonid taxonomy, and ongoing efforts are underway to describe additional species in the family [36], [37], which appears to be underestimated (F. Marqes, unpublished data; M. R. de Carvalho, pers. comm.). The cestode taxonomy was also relatively unresolved at the time hypotheses on the origin of potamotrygonids and their parasites were first proposed. Prior to 1981, only eight species of cestodes had been described from potamotrygonids [38]–[43]. Since that time, several taxonomic studies, including descriptions of new species and genera, helped strengthen the taxonomic backbone for the cestode parasites of potamotrygonids [27]–[29], [44]–[52]. Despite these efforts, the present taxonomic status of the lineages in this potamotrygonid/parasite system remains far from meeting the criteria required to provide an accurate estimate of patterns and processes involved on the historical associations between hosts and their parasites [13], namely, robust circumscriptions of species are still needed.

Our intent with this contribution is to refine the systematics of one component of this host/parasite system, that is, to investigate diversity of lineages of *Rhinebothrium* found in potamotrygonids using morphological and molecular data. To date, there are more than 40 species of *Rhinebothrium* described [53], [54]. Of these, three species are parasites of potamotrygonids (*Rhinebothrium paratrygoni* Rego and Dias, 1976, and the more recently described *Rhinebothrium copianullum* Reyda, 2008, and *Rhinebothrium paranaensis* Menoret and Ivanov, 2009). Several of these are circumscribed by limited data. The goal of this study was to obtain a better understanding of species boundaries within this genus. The strategy employed was to perform a widespread sampling effort, represented by approximately 900 worms obtained from most of the nominal species presently recognized for potamotrygonids, in two major river systems of South America, and to investigate species boundaries with both morphological and molecular data. In doing so, we address the patterns of distribution of what we recognize as putative freshwater species within *Rhinebothrium*, with emphasis on the patterns of host specificity observed among these lineages.

## Materials and Methods

### Freshwater stingray specimens

**Figure 1:**
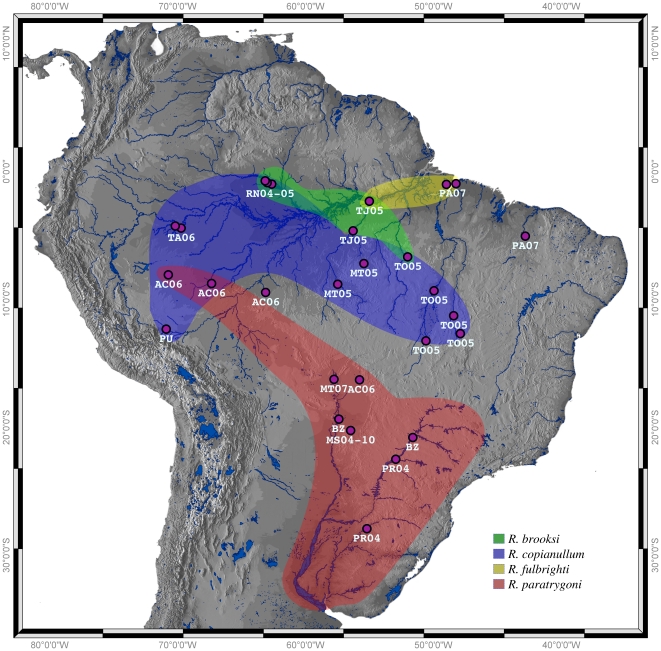
Collection sites of Potamotrygonid stingrays in the Amazon and La Plata river basins, South America. Color shapes indicate approximate distributions of *Rhinebothrium* species. Details of field codes shown here are given in Table S1.

Freshwater stingrays were collected from multiple localities (with designated field codes, see Table S1), and in some cases, during more than one year, throughout the Amazon and La Plata River basins in South America (see Fig. 1). These localities represent almost all drainages systems of South America from which potamotrygonids have been historically reported [23], [24] and/or included type localities for tetraphyllideans found in potamotrygonids; e.g., two different collections at the Salobra River (Mato Gross do Sul State, Brazil), the river referred to as the type locality of *R. paratrygoni*. Stingrays were collected with hand-held spears, spear guns, or with the use of hand-held lines, landlines, or long-lines using small teleosts as bait, in conjunction with local fisherman. All collections in Brazil were conducted following the guidelines of a collecting permit issued to F. P. L. Marques, J. N. Caira, and F. B. Reyda by the Environmental Ministry of the Brazilian Federal Government (IBAMA Proc. no. 02001.007961/2002–31 issued on January 8, 2003) and those issued to F. P. L. Marques (IBAMA no. 087/94–DIFAS of February 16, 1995; 006/96–DIFAS of January 19, 1996; no. 015/2004 of January 13, 2004; 083/05–DIFAS of July 15, 2005; 071/06–DIFAS of June 23, 2006 10008–1 of January 26, 2009; and 24451–1 of July 08, 2010). All collections in Peru were done following the guidelines of collecting permits issued to F. B. Reyda by the Peruvian Ministry of Fisheries in Lima (Permits CE–00152002 and CE–00036001). Stingrays were examined for parasites following euthanization by cranial concussion under the authorization of University of Connecticut IACUC protocol No. C010 0202.

Following examination for parasites, stingrays were fixed in formalin diluted from 40% to 4% with 0.6% saline, stored for several days, and subsequently transferred to 70% ethanol. The majority of stingray specimens collected were deposited at the Museu de Zoologia da Universidade de São Paulo, Brazil (MZUSP). Stingrays were identified based on de Carvalho *et al.* [19] and Rosa [18] by F. P. L. Marques in conjunction with M. de Carvalho (Universidade de São Paulo, Brazil). Unidentified species were assigned either by regional common names (e.g. *Potamotrygon* sp. (cururu)) or by a code in reference to the drainage in which the morphotype(s) was (were) found (e.g., *Potamotrygon* sp. (tpj1) and (tpj2), since we found two potentially undescribed forms in Rio Tapajós). Images of each stingray specimen from which cestodes were collected are available on-line (see Marques & Domingues [55]: http://www.ib.usp.br/hpc/hpc_index.htm) and can be searched in the database using the field code for each locality that is provided in Table S1.

### Collected cestode specimens

The spiral intestine of each stingray was removed, opened with a mid-ventral incision, and examined for parasites with a dissecting microscope. Cestodes and other parasites encountered were placed in 95 or 96% ethanol, or in formalin diluted from 40% to 4% with 0.6% saline. After several days specimens were transferred to 70% ethanol for storage. In addition, the spiral intestine of the majority of each stingray collected was secondarily examined in the laboratory. Cestode specimens prepared as whole mounts for light microscopy were hydrated in a graded ethanol series, stained in Delafield's or Harris's hematoxylin, dehydrated in a graded ethanol series, cleared in methyl salicylate, and mounted on glass slides in Canada balsam. Information on cestode genera other than *Rhinebothrium* will be provided in other ongoing studies.

Specimens for histological sectioning were embedded in paraplast and sectioned at 8 µm intervals using an Olympus CUT4060 retracting rotary microtome. Sections were mounted on glass slides flooded with 2.5% sodium silicate and dried on a slide warmer for 4 to 8 h. Cross sections of mature proglottids and longitudinal sections of scoleces were prepared for each species described here. Sections were stained with Delafield's hematoxylin and eosin (H&E) according to conventional techniques. A portion of each worm sectioned was prepared as a whole mount, as above, and kept as a voucher.

Scoleces of 1 or more specimens of each cestode species, and multiple free proglottids of each species, were prepared and examined with scanning electron microscopy (SEM). Each scolex prepared for SEM was cut from its strobila with a scalpel, and the strobila was prepared as a whole mount, as above, and kept as a voucher (hologenophores, sensu Pleijel *et al* [56]. Specimens to serve as vouchers for the free proglottids that were prepared for SEM were obtained as free proglottids that could be identified as conspecific from the same host individual (paragenophores, sensu Pleijel *et al* [56]. All SEM specimens were hydrated in a graded ethanol series, transferred to 1.5% osmium tetroxide overnight, dehydrated in a graded ethanol series, and placed in hexamethyldisilizane (HMDS, Ted Pella Inc., Redding, CA) for 15 min. They were allowed to air dry and were subsequently mounted on carbon tape and grounded with carbon paint on aluminum stubs. They were sputter-coated with ∼200–300 Å of gold/palladium and examined with a LEO/Zeiss DSM 982 Gemini Field Emission Scanning Electron Microscope.

A portion of each DNA sequenced cestode specimen that was sequenced (see below) was prepared as a whole mount, as above, and kept as a molecular voucher. These molecular voucher specimens can be considered hologenophores (sensu Pleijel *et al* [56]) because they are same organism that was used for the molecular work conducted. Each hologenophore was deposited in a museum and given individual numbers (see Table S2).

All cestode specimens prepared as whole mounts, as histological sections, as SEM specimens, and as vouchers, were deposited at MZUSP, the United States National Parasite Collection, Beltsville, Maryland, U.S.A. (USNPC) or the Lawrence R. Penner Parasitology Collection, University of Connecticut, Storrs, Connecticut, United States (LRP).

### Museum cestode specimens

The holotype and paratypes of *R. paratrygoni* were examined at the Colecão Helmintologica do Instituto Oswaldo Cruz, Rio de Janeiro, Brazil (CHIOC). Voucher specimens of *R. paratrygoni* from MZUSP, from USNPC, and from the Harold W. Manter Laboratory of Parasitology, University of Nebraska State Museum, Lincoln, Nebraska, U.S.A. (HWML), were also examined. Paratypes of *R. copianullum* from LRP were examined. In addition, paratypes of *R. paranaensis* from the Colección Parasitológica, Museo Argentino de Ciencias Naturales, Buenos Aires, Argentina (MACN–Pa) were examined.

### Morphological analyses of cestodes

An ocular micrometer was used on a Zeiss Axioscope 2, or an Olympus CH2 to measure *Rhinebothrium* specimens that were prepared as whole mounts, including whole mounts that served as vouchers of worms from which molecular sequence data were generated. Only specimens possessing proglottids that were mature (i.e., with distinctly formed male and female genitalia) or further developed (e.g., with sperm-filled vas deferens and atrophied testes) were measured in this study. Measurements of all genitalia were taken from terminal proglottids, unless terminal proglottids were further developed, in which cases testes measurements were only taken from subterminal mature proglottids. Mature or gravid free proglottids that could be assigned to a particular *Rhinebothrium* species with confidence were also measured. Measurements are presented as ranges, with the mean, standard deviation, number of specimens examined and number of measurements taken given in parentheses. All measurements are in micrometers unless otherwise specified. Line drawings were prepared with the aid of a camera lucida. Terminology used for microthrix types follows that of Chervy [57], and terminology used to describe bothridia shape follows the nomenclature of plane shapes provided by Clopton [58].

### Nomenclatural acts

The electronic version of this document does not represent a published work according to the International Code of Zoological Nomenclature (ICZN), and hence the nomenclatural acts contained in the electronic version are not available under that Code from the electronic edition. Therefore, a separate edition of this document was produced by a method that assures numerous identical and durable copies, and those copies were simultaneously obtainable (from the publication date noted on the first page of this article) for the purpose of providing a public and permanent scientific record, in accordance with Article 8.1 of the Code. The separate print-only edition is available on request from PLoS by sending a request to PLoS ONE, Public Library of Science, 1160 Battery Street, Suite 100, San Francisco, CA 94111, USA along with a check for $10 (to cover printing and postage) payable to “Public Library of Science”.

In addition, this published work and the nomenclatural acts it contains have been registered in ZooBank, the proposed online registration system for the ICZN. The ZooBank LSIDs (Life Science Identifiers) can be resolved and the associated information viewed through any standard web browser by appending the LSID to the prefix “http://zoobank.org/”. The LSID for this publication is: urn:lsid:zoobank.org:pub:345F527C-4513-4424-9D62-28109E64B25A.

### DNA extraction, gene amplification and sequencing of cestode specimens

Specimens for which molecular data were generated included *Rhinebothrium* and *Rhinebothroides* specimens from potamotrygonids and selected marine rhinebothriine specimens provided by J. N. Caira, K. Jensen, and C. Healy. All specimens that were sequenced are listed in Table S2. Portions of cestodes were allowed to air-dry, and prepared for genomic DNA extraction, using 1 of the following 3 protocols. Tissue was (1) Incubated in 18 µl Worm Lysis Buffer (100 µl 0.5 M KCl; 200 µl 50 mM Tris; 50 µl 50 mM MgCl2; 4.5 µl NP–40; 4.5 µl Tween 20; 641 µl MilliQ water) and 2 µl Proteinase K for 20 minutes at 65°C followed by incubation at 95°C for 10 minutes, or (2) Processed with the Nucleospin extraction kit following the protocols outlined in the accompanying handbook, with the exception that the final elution volume was 30 µl rather than 100 µl, or (3) Incubated with 2× CTAB buffer at 37°C for ≥8 hours, and subsequently processed with a conventional chloroform-phenol extraction protocol.

PCR was performed on a 572 bp region of the COI gene using the forward primers SEAN-1 (5′-TTT ACT TTG GAT CAT AAG CG-3′) or nLCO (5′-TTT ACT YTR GAY CAT AAG CGT-3′), and the reverse primers BEN-1 (5′-RGT ACC AAA AAA CCA AAA CA-3′), or BEN-5 (5′-AAG CAG AAC CAAA TTT ACG ATC-3′), or SEAN-2 (5′- AAG CAG AAC CAA ATT TAC GAT-3′). Thermal cycles were as follows: initial denaturation for 2–5 min at 94°C, followed by 35 cycles of 30 secs–1 min at 94°C, 40 secs–1 min at 48.5–50°C, and 1 min at 72°C, followed by a final extension of 5–7 min at 72°C. PCR products were purified either using an Ampure™ kit or Qiagen™ columns. Products were subsequently either re-amplified or cycle-sequenced directly from forward, reverse, and, in some cases, internal strands, using ABI Big-Dye™ chemistry, cleaned with sephadex beads, and sequenced on an ABI automated sequencer.

Contiguous sequences were assembled and edited using Sequencher™ 4.6. or the package Consed/PhredPhrap [59]–[62]. Sequences were aligned using Clustal W [63] spawned from BioEdit [64]. After alignment, sequences were checked for stop codons using the DNA to Protein Translation online resource by Bikandi *et al.* [65] and all sequences were trimmed so that the first base corresponded to the first codon position.

### Phylogenetic analyses

The analytical protocol started by creating 3 partition schemes based on codon positions of COI. In the first partition scheme each codon position was treated separately; hereafter referred as (1)(2)(3); for the second partition scheme first and second positions were considered a separated block from the third position (1,2)(3); and finally, the third partition scheme considered all positions comprised a single data block (1,2,3). Since model-based methods of phylogenetic inference require the choice of substitution models, which must be selected in a statistically rigorous manner [66], we submitted each partition scheme to model-selection software JModelTest [67]. In order to avoid the use of unsupported models, which can affect the outcome of phylogenetic analysis and in some cases, generate different tree topologies ([66], and references therein). We selected different models of nucleotide substitution using the AICc (corrected Akaike information criterion) and BIC (Bayesian information criterion) model selection criteria [68]. For each run in JModelTest, we estimated the optimal substitution model from 88 possibilities (11 substitution schemes + F + I + Г) using ML optimized topologies. AICc and BIC converged on identical substitution models (*i.e.*, TIM2+I+Г) for partitions (1,2) and (1,2,3). However, for partitions (1), (2), and (3), AICc selected the models K80+Г, JC+I, and TIM2+I+Г, whereas the BIC favored the models TIM1+Г, TIM3+I, and TIM3+I+Г, respectively.

Phylogenetic analyses based on COI fragments were performed under two optimality criteria. We estimated the maximum likelihood (ML) topology using the program GARLI-PART (*ver.* 0.97.r737; [69], [70]. This application allows partitioning of data into subsets, each of which may be assigned to separate evolutionary models, with parameters independently estimated. Five runs were conducted based on favored substitution models under each alternative model-selection method (*i.e.*, AICc and BIC). For each run, we performed 5,000 independent search replicates (*searchreps* = 5000), using different subset rates (*linkmodels* = 0 and *subsetspecificrates* = 1) – when applicable, and remaining default parameters of GARLI-PART configuration file. For the ML analyses, nodal support was inferred by bootstrap proportions after 5,000 bootstrap replicates with two independent search replicates each (*bootstrapreps* = 5000 and *searchreps* = 2). In addition, we estimated the tree topology using Maximum Parsimony (MP) with the program PAUP* (ver. 4.0b10; [71]) using tree bisection reconnection branch swapping during heuristic searches of tree space on 1,000 randomly constructed starting trees (*hsearch nreps* = 1000 *addseq* = *random*). Uncorrected patristic distances were obtained using PAUP* with default options. Additional measures and summary statistics were obtained using DNAsp (version 5.10.01, [72]). Datasets, configuration files for GARLI-PART, and resulting tree files have been deposited in TreeBASE (S11002).

## Results

### Phylogenetic analyses

**Figure 2:**
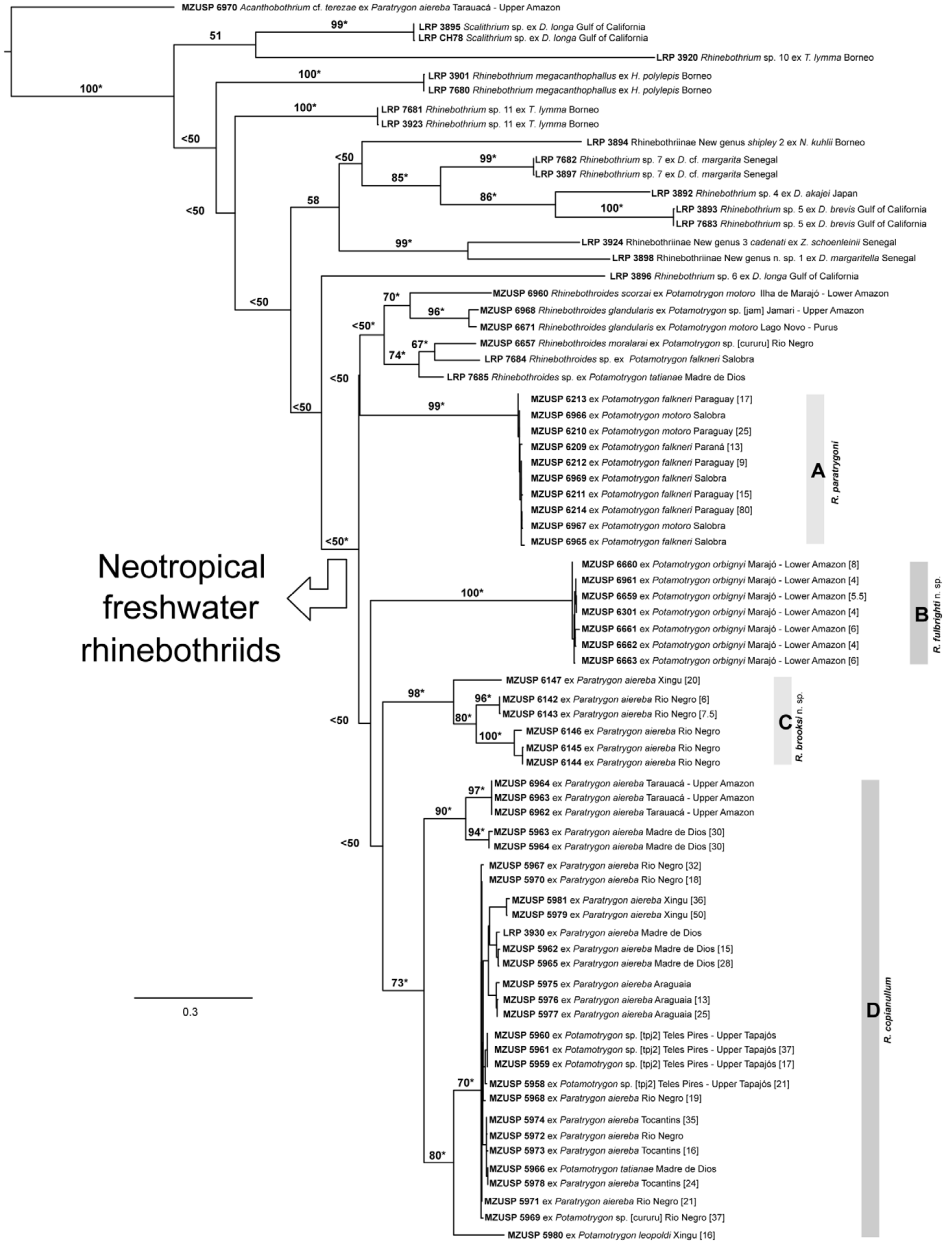
Best-scoring ML tree (−lnL = 7193.942769) based on COI data from marine and freshwater rhinebothriids. Numbers on internal branches denote nodal support as inferred by Bootstrap Proportions based on 5,000 replicates. *, indicates nodes recovered during phylogenetic analysis under parsimony. Numbers between square brackets in front of terminals represent total length in millimeters for those specimens measured. Scale indicates expected number of substitution per site.

After completing the ML analyses for 5 distinct substitution models for 3 distinct codon partition schemes, all likelihood scores were used to select the partition/substitution model that minimizes AICc and BIC scores (Table S3). According to our results, AICc and BIC converged on the same partition/substitution model (Table S3) favoring the partition scheme in which the substitution model TIM2+I+Γ was applied to the 1^st^ and 2^nd^ codon positions and the model TIM3+I+Γ was applied to the 3^rd^ codon position. These substitution models had been selected by the BIC model selection criterion during the model selection phase. The resulting topology for this partition scheme and substitution model is presented in Fig. 2.

The phylogenetic analysis using the Maximum Parsimony optimality criterion (MP) generated 20 most parsimonious topologies each 1772 steps. A strict consensus tree recovered most of the nodes present on the ML topology (Fig. 2, nodes noted with “*”). With respect to freshwater rhinebothriids, clades marked A–D in the ML topology in Fig. 2 were also monophyletic groups in the MP topology. Differences between the two topologies were that in the MP tree, specimens from clades A and B nested as sister clades, but did not in the ML, and the relationships between C and D were unresolved in the MP, unlike in ML, in which they were sister groups (Fig. 2). Since our main concern here is to recognize monophyletic assemblages of haplotypes for freshwater lineages of *Rhinebothrium*, we will not address the phylogenetic pattern recovered for marine lineages. Given the low taxonomic representation for marine species of *Rhinebothrium* and the use of a single locus to infer a species tree, we find that it is premature to discuss the phylogenetic relationships among all lineages of this genus. All trees generated under both optimality criteria are available in TreeBase (S11012).

In both analyses, the freshwater stingray rhinebothriids formed a clade, although poorly supported by bootstrap values. Within this clade, five clades of rhinebothriids were recognized. One of these clades consisted of species of *Rhinebothroides* (Fig. 2). The other 4 clades consisted of haplotypes of specimens that morphologically conform to the diagnosis of *Rhinebothrium* as emended herein. Each of these was considered to represent putative species, and was supported by combinations of morphological features (see below). The phylogenetic relationships among these clades are ambiguous; nodes defining nested sets either had low bootstrap support in the ML analysis (Fig. 2), or collapsed in the MP analysis. However, except for the node for *Rhinebothroides*, all of the clades were strongly supported in our analyses. Two of the four clades (A and D) in Fig. 2 correspond to previously known species that are redescribed below, and the two other clades (B and C) correspond to new species described below.

Below, we characterize the morphological features, host associations, and biogeographic distributions of each of the putative species represented by each clade of haplotypes of freshwater specimens of *Rhinebothrium*, and use this information to revise the taxonomy of the group.

Order RHINEBOTHRIIDEA

Genus *Rhinebothrium* Linton, 1890

*Rhinebothrium paratrygoni* Rego and Dias, 1976. Redescription.

Figs. 3, 4, 5

**Figure 3:**
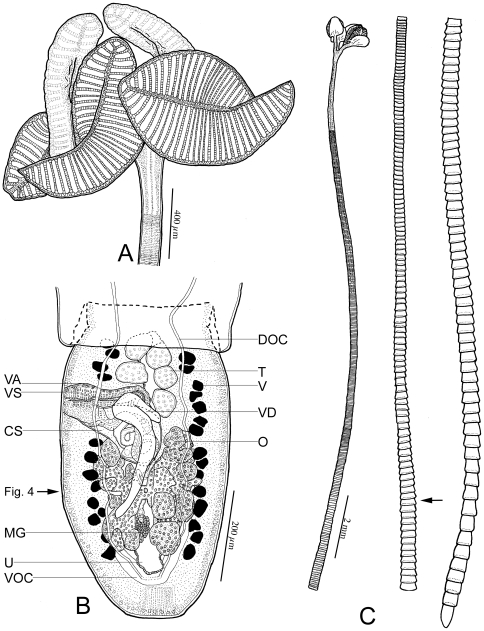
Line drawings of *Rhinebothrium paratrygoni* Rego & Dias, 1976 collected from the type locality. A. Scolex of voucher (MZUSP 6214). B. Terminal mature proglottid of voucher (MZUSP 6214). Vas deferens is above cirrus sac. Arrow indicates location of section shown in Figure. 4. C. Whole worm of voucher (MZUSP 6260k), illustrated in 3 fragments, from left to right: Anterior, middle and posterior. Arrow indicates anterior most mature proglottid. *Abbreviations*: CS, Cirrus sac; DOC, Dorsal Osmoregulatory canal; MG, Mehlis' gland; O, Ovary; T, Testes; U, Uterus; V, Vitellaria; VA Vagina; VD, vas deferens; VS Vaginal sphincter; VOC, Ventral Osmoregulatory canal.

**Figure 4:**
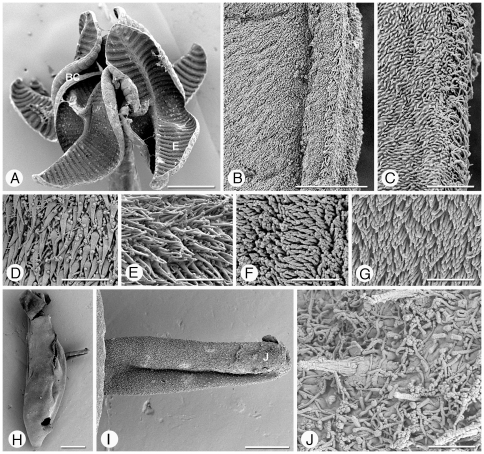
Scanning electron micrographs of *Rhinebothrium paratrygoni*. Scolex, Figures A–G. A. Scolex. B. Small letter indicates locations of details shown in B–C, E. Proximal surface of rim of bothridium. C. Proximal bothridial surface adjacent to bothridial rim. D. Proximal bothridial surface. E. Transverse septum on distal bothridial surface. F. Stalk surface. G. Strobila surface. Cirrus, Figures H–J. H. Free proglottid with everted cirrus. I. Everted cirrus. Small letter indicates location of detail shown in J. J. Coniform spinitriches and capilliform filitriches on distal portion of cirrus. Scale bars: A, 200 µm; B, 10 µm; C–G, 2 µm; H, 200 µm; I, 50 µm; J, 2 µm.

**Figure 5:**
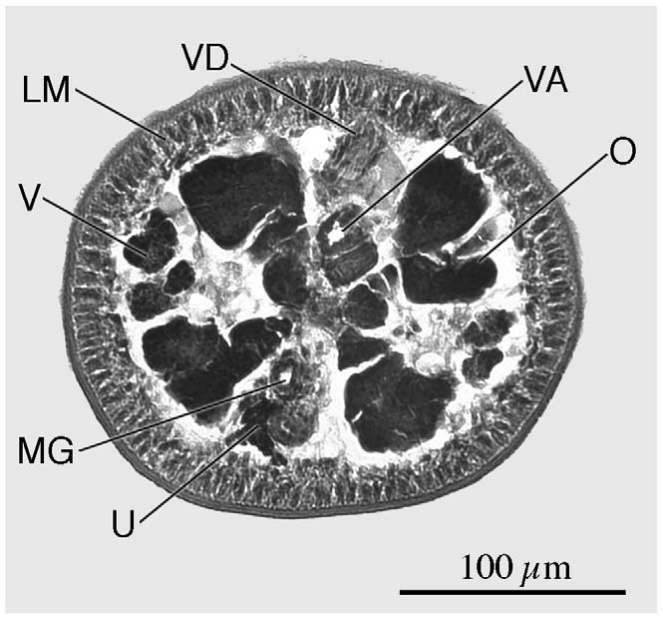
Cross-section through mature proglottid of *Rhinebothrium paratrygoni* at level of ovarian isthmus. *Abbreviations*: LM, Longitudinal muscles; MG, Mehlis' gland; O, Ovary; U, Uterus; V, Vitellaria; VA Vagina; VD, vas deferens.

*Redescription* (based on the Holotype, CHIOC 31.213b, 4 paratypes CHIOC 31.213a, 31.213 c–e, and newly collected vouchers consisting of 57 whole mounts of mature worms, including 7 molecular vouchers measured, 2 free mature proglottids, 8 free gravid proglottids, 10 pairs of proglottids en copula, cross sections of 1 strobila, longitudinal sections of 1 scolex, 6 scoleces and 4 proglottids prepared for SEM): Worms (Fig. 3C) euapolytic, or rarely apolytic, very craspedote, 8–80 (32±18; n = 57) mm long, greatest width 780–2250 (1189±356; n = 39) at level of scolex; 266–1060 (610±196; n = 52) proglottids per worm. Scolex (Figs. 3A and 4A) consisting of scolex proper bearing 4 stalked bothridia. Bothridia fusiform-shaped, lacking constriction at center, with muscular rims, 780–1210 (1000±140; n = 22; *n* = 23) long, 350–670 (477±84; n = 19; *n* = 20) wide, maximum width at or slightly anterior to middle, divided by 31–35 (33±1; n = 19; *n* = 22) transverse septa and 1 medial longitudinal septum into 63–71 (66±2; n = 19; *n* = 22) transversely oriented loculi. Medial longitudinal septum extending from posterior margin of anteriormost loculus to posterior margin of bothridium. Anteriormost loculus single, 35–67 (49±8; n = 33; *n* = 43) long, 50–87 (71±10; n = 34; *n* = 46) wide; posteriormost loculi double, 37–60 (49±8; n = 16) long, 25–42 (35±5; n = 16) wide. No marginal loculi observed. Stalks 100–650 (338±137; n = 36; *n* = 55) long, 60–200 (117±33; n = 35; *n* = 54) wide, attached to bothridium at middle or slightly posterior to middle of bothridium. Cephalic peduncle lacking; neck varying in length.

Proximal surfaces of bothridia, except for the margins, covered with acicular filitriches and coniform spinitriches (Fig. 4D); bothridial margin (Fig. 4B), bearing only capilliform filitriches (Fig. 4C). Distal surfaces of bothridia with acicular filitriches and coniform spinitriches throughout, including surfaces of longitudinal and transverse septa (Fig. 4E). Stalks (Fig. 4F), neck, and strobila (Fig. 4G) bearing capilliform filitriches.

Strobila: Greatest proglottid width 210–850 (439±131; n = 50) near posteriormost proglottids. Majority of proglottids wider than long; posteriormost 1–8 (3±2.2; n = 47) proglottids longer than wide; mature proglottids 3–90 (41±27; 38) in number, gravid proglottids 0–1 (0.05±0.2; n = 38) in number.

Terminal proglottid (Fig. 3B) 280–830 (525±142; n = 52) long, 180–410 (294±57; n = 52) wide, length to width ratio 0.9–3.6 (1.8±0.6; n = 51). Genital pores marginal, irregularly alternating, 63–84% (72±5.1; n = 47) of proglottid length from posterior end. Testes irregularly oval, 40–100 (64±10; n = 47; *n* = 117) long by 30–75 (45±9; n = 76; *n* = 116) wide, all in primary field, 4–9 (5±1; n = 54; *n* = 124) in total number, 1–2 layers deep, in as many as 3 irregular columns, extending from near anterior margin of proglottid to level of genital pore. Vas deferens in terminal proglottids coiled, spanning from posteriormost testes posteriorly to ovarian isthmus, entering center of anterior margin of cirrus sac. Cirrus sac elongate oval or triangular, slender in subterminal mature proglottid, extending medially to, or well past, midline of proglottid, extending posteriorly to anterior ovarian margin or to ovarian isthmus, containing coiled cirrus. Cirrus sac in terminal proglottid 105–225 (164±27; n = 49) wide by 70–172 (114±23; n = 49) long. Everted cirrus (Figs. 4H and 4I) 270–390 (340±56; n = 4) long, 60–70 (68±5; n = 4) wide, covered with capilliform filitriches and with coniform spinitriches (Fig. 4J); coniform spinitriches of cirrus 2–2.5 (2.2±0.3; n = 7) in length, 0.7–0.85 (0.78±0.1; n = 2) in width, Vagina thick–walled, sinuous, varying in width along its length, with darkly staining cells, extending laterally from common genital atrium, then posteriorly along medial line of proglottid to ootype, with sphincter. Proximal portion of vagina slightly expanded. Ovary near posterior end of proglottid, lobulated, H-shaped in frontal view, tetra-lobed in cross section (Fig. 5), symmetrical, 110–325 (195±54; n = 49) long, maximum width 110–270 (183±39; n = 46); ovarian isthmus located at or slightly anterior to mid-point of ovary. Anterior margin of ovary 40–213 (96±33; n = 42) short of genital pore. Mehlis' gland posterior to ovarian isthmus, 50–75 (66±7; n = 8) long and 40–50 (45±4; n = 8) wide. Vitellarium follicular; vitelline follicles 10–42 (26±8; n = 46; *n* = 114) long, 6–30 (17±6; n = 30; *n* = 114) wide, arranged in 1 dorsal and 1 ventral column on each side of proglottid, extending from level of anteriormost testes to slightly posterior to the ovary, interrupted dorsally by the cirrus sac and vagina. Uterus ventral, sacciform, extending from posterior margin of ovary to near anterior margin of proglottid.

Free mature proglottids 1000–1270 (1135±191; n = 2) long, 410–500 (455±64; n = 2) wide. Free gravid proglottids 1100–1800 (1404±240; n = 8) long, 340–820 (529±136; n = 8) wide. Proglottids of equal or unequal length observed en copula.

### Taxonomic summary

#### Synonym

*Rhinebothrium paranaensis* Menoret and Ivanov, 2009.

#### Material examined and material deposited

Holotype and 4 paratypes of *R. paratrygoni* (CHIOC 31.213b, 31.213a, and 31.213 c–e respectively). Voucher specimens of *R. paratrygoni*: HWML 21010, 21016, 34095; USNPC 75712; MZUSP 5866–5909. Paratypes of *R. paranaensis* (MACN–Pa nos. 478/2, 478/3, and 478/5). Voucher specimens of *R. paratrygoni* examined and deposited as part of the current study: Fifty whole mounts (MZUSP 6214, 6250a, 6252, 6253a, 6254a–b, 6256b, 6258a, 6258c, 6259b, 6260d–e, 6260i–o, 6261d, 6263h–i, 6264a, 6265a, 6265f, 6266a–b, 6267h, 6268a, 6269; LRP 7657, 7659–7661, 7663–7667, 7670; USNPC 104716, 104718–104720, 104721 (4 slides), 104723, 104725), two free mature proglottids (MZUSP 6259e, 6265j), eight free gravid proglottids (6259a, 6263f–g, 6267b, 6267d–e; LRP 7668; USNPC 104724), ten pairs of proglottids en copula (MZUSP 6260a, 6261a, 6263a, 6254e, 6258b; LRP 7662, 7667; USNPC 104718, 104721–104722), six scoleces and four free proglottids prepared for SEM (MZUSP 6237–6243, 6257, LRP 7658, USNPC 104717, respectively), cross sections of one strobila and longitudinal sections of one scolex of one worm ((MZUSP 6197a–q (including vouchers)), and ten voucher specimens (i.e., hologenophores) of sequenced worms (MZUSP 6209–6214, 6965–6967, 6969 for GenBank Nos. JF80684–JF80693).

#### Type host

An unidentified species of *Potamotrygon*.

#### Additonal hosts

*Potamotrygon falkneri*, *Potamotrygon motoro*, *Potamotrygon brachyura*, *Potamotrygon histrix*, *Potamotrygon* sp. (tar1), and *Potamotrygon* sp. (tar2).

#### Type locality

Rio Salobra, Mato Grosso do Sul State, Brazil.

#### Additional localities

Rio Salobra, Paraguay sub-basin, La Plata Basin, at Miranda, Distrito de Salobra, Mato Grosso do Sul State, Brazil (Lat: 20°12′36″S Long: 56°30′0″W); Rio Apa, Paraguay sub-basin, La Plata Basin, at Bela Vista, Mato Grosso do Sul State, Brazil (Lat: 22°6′36″S Long: 56°30′36″W); Rio Paraná, Paraná sub-basin, La Plata Basin, at Jupiá, Distrito de Três Lagoas, Mato Grosso do Sul State, Brazil (Lat: 20°47′24″S Long: 51°38′60″W); Rio Paraná, Paraná sub-basin, La Plata Basin, at Porto Primavera, São Paulo State, Brazil (Lat: 22°28′12″S Long: 52°57′36″W); Padre Inácio, Paraguay sub-basin, La Plata Basin, at Cáceres, Mato Grosso State, Brazil (Lat: 16°14′28″S Long: 57°47′9″W); Rio Mutum, Paraguay sub-basin, La Plata Basin, at Barão de Melgaço, Mato Grosso State, Brazil (Lat: 16°17′56″S Long: 55°48′13″W); Rio Uruguay, Uruguay Basin, at Porto Xavier, Rio Grande do Sul State, Brazil (Lat: 27°53′60″S Long: 55°13′12″W); Colastine River, Rio La Plata or Paraná Basin, at Santa Fé, Argentina (Lat: 31°40′S, 60°46′W); Lago Novo, Juruá sub-basin, Amazon Basin, at Boca do Acre, Amazonas State, Brazil (Lat: 8°44′46″S Long: 67°22′52″W); Lago Arara, Juruá sub-basin, Amazon Basin, Acre State, Brazil (Lat: 8°4′6″S Long: 70°43′5″W).

#### Site of infection

spiral intestine.

#### Additional vouchers deposited

Cross sections of five strobilae (MZUSP 6203a–c, 6205a–b, 6206a–c, 6207a–d, 6208a–c), longitudinal sections of three scoleces (MZUSP 6201a–d, 6202a–c, 6204a–c), and 167 whole mounts (MZUSP 6198a–e, 6199, 6215–6236, 6244–6249, 6250b–d, 6251a, 6253d, 6254f, 6254h–p, 6255a–b, 6256a, 6256c–k, 6258f–p, 6259f–u, 6260h, 6260w–z, 6260za–zo, 6261e–f, 6263b–e, 6263j–p, 6264d–f, 6265b–e, 6265g–i, 6265k–o, 6267a, 6267c, 6267i–n, 6268d, 6270–6273, 6274a–d, 6275a–b, 6276a–b, 6277a–c, 6278a–c, 6279–6280, 6281a–c).

### Remarks

Confusion about the concept of *Rhinebothrium paratrygoni* Rego and Dias, 1976 spawns from the brevity of its original description, as well as from the poor understanding of the morphological variability within lineages of freshwater species of *Rhinebothrium*. In the original description, Rego and Dias [41] stated that the bothridia were bi-lobed and that one of the lobes was wider than the other. However, in at least one of the paratypes (CHIOC 31.213c) the bothridia consist of an anterior and a posterior portion, and are not bi-lobed, and the maximum bothridial width is at, or near, the middle of the bothridium. These authors also stated that the vagina enters the genital atrium posterior to the cirrus sac, but the vagina in all of the museum specimens examined enters the genital atrium anterior to the cirrus sac. Among the type material used on the original description, the holotype is the only complete specimen in the series, and it is immature, the paratypes are incomplete and/or immature. As a consequence, most measurements in the original description (*i.e.*, worm length, scolex width, bothridia length and width, proglottid length and width, cirrus sac length and width, and ovary length) are poor estimators of the morphometric attributes of mature *R. paratrygoni*. Nonetheless, based on the type material, *R. paratrygoni* possesses 100 s of proglottids (e.g., the holotype, although immature, possesses 682 proglottids); lacks a cephalic peduncle, possesses 4 pedunculated bothridia, each divided by a single longitudinal and multiple transverse septa into ∼71 facial loculi, including a single anteriormost loculus and a pair of posteriormost loculi, possesses craspedote strobila and proglottids with 5–6 testes and a cirrus with spinitriches ∼2 long. However, no other morphological attributes, morphometric and/or meristic, can be assigned unequivocally to this name.

Despite the limitation from the type material and the original description, Menoret and Ivanov [73] recently described *Rhinebothrium paranaensis* Menoret and Ivanov, 2009 from *Potamotrygon falkneri* from a tributary of the Paraná River in Argentina. Menoret and Ivanov [73] provided justification for *R. paranaensis* as a novel species mainly based on morphometric attributes taken solely from the original description of *R. paratrygoni*. For example, with respect to *R. paratrygoni*, the new species differed in total length (47.8–77.9 vs. 23 mm), scolex width (900–1,400 vs. 870), proglottid number (800–1,014 vs. 682), cephalic peduncle length (190–310 vs. 150), and cirrus sac width (62–140 vs. 46). It comes as no surprise that, as examination of a considerable number of specimens, some of which were collected from the type locality of *R. paratrygoni* (Salobra River in the Paraná Basin, Mato Grosso do Sul State, Brazil; see Table S1) and including the type material of both nominal species, the morphological differences between these two species reported by Menoret and Ivanov [73] were not supported. Most of the measurements provided for *R. paranaensis* by Menoret and Ivanov [73] were found to fall within the ranges of the measurements provided above in the redescription of *R. paratrygoni* that is based on additional material. Examination of several paratypes of *R. paranaensis* (MACN–Pa nos. 478/2, 478/3, and 478/5) revealed that they are conspecific with the specimens included above in the redescription of *R. paratrygoni* because they possess scoleces with eliptoid- or diamond-shaped bothridia that lack a constriction at the center, 100's of craspedote proglottids that are wider than long and cirrus sac possessing a cirrus with spinitriches ∼2 long. *Rhinebothrium paranaensis* is therefore considered a junior synonym of *R. paratrygoni*. Differences between our redescription of *R. paratrygoni* and *R. paranaensis* that were seen were considered to represent intra-specific variation. These include anterior loculus length (35–67 vs. 17–40), mature terminal proglottid length (280–830 vs. 203–540), and cirrus sac width (70–172 vs. 62–140).

Some of the morphological characters used by Menoret and Ivanov [73] to distinguish between these species also deserve some comment. Menoret and Ivanov [73] described *R. paranaensis* as having two irregular columns of testes, but the testicular fields in the paratypes of *R. paranaensis* we examined (e.g., MACN–Pa nos. 478/2) could be considered as having up to three columns. What Menoret and Ivanov [73] referred to as a cephalic peduncle in their description is more appropriately referred to as a neck, i.e., an elongated germinative zone posterior to the scolex in which proglottids are produced (Caira & Jensen 2011 [74]; http://sites.google.com/site/tapewormpbi/) rather than a narrow muscular extension of the scolex that supports the scolex proper. Menoret and Ivanov [73] stated that *R. paranaensis* lacks a vaginal sphincter, but we observed prominent muscular bands near the genital atrium in the paratypes of *R. paranaensis* (e.g., MACN–Pa nos. 478/2), and in the *R. paratrygoni* specimens here, that could be considered a vaginal sphincter.

In the context of current tetraphyllidean taxonomy, it could be argued that the amount of variation seen here might imply the existence of hidden distinct evolutionary lineages within the revised concept of *R. paratrygoni*. One should, however, consider that the present understanding of morphological variability within tetraphyllidean species is generally based on limited material. A non-exhaustive survey of the tetraphyllidean taxonomic literature of the last 20 years revealed that the number of specimens on which redescriptions or descriptions is based averages 18, with a median of 14 and a range from 1–108 specimens. In most cases these specimens are from isolated locations and low numbers of hosts (Marques, unpubl. data based on 46 publications). On the other hand, our understanding of the morphological variation of *R. paratrygoni* is based on measurements of 57 mature worms, and observation of 167 additional specimens, SEM images, and histological data, from a pool of samples obtained from 31 infected stingrays (of 217 examined) from the La Plata River System, as well as from two localities in the Amazon River System.

Despite the fact that our molecular analysis was based on an analysis of data from only a single locus, we think some properties of Clade A (Fig. 2) can be used to justify our concept of *R. paratrygoni*. Clade A is highly supported, as inferred by bootstrap values (99) – and indeed represents one of the longest branches leading to a cluster of haplotypes. This clade is comprised of haplotypes of 10 specimens, nine from the type locality of *R. paratrygoni*, and one from the Paraná River. These haplotypes exhibited low nucleotide diversity (π = 0.00778) and, hence, narrow uncorrected pairwise patristic distances variation (ranging from 0 to 0.01889), suggesting that they are cohesive. Despite the molecular cohesion observed for COI in Clade A, the specimens representing the haplotypes ranged greatly in total length, from 9 to 80 mm (See Fig. 2, Clade A), and hence number of proglottids (data not shown). We were not able to recover any cladistic structure within this clade correlated with worm size and/or number of proglottids and, except for the differences in theses traits, all worms exhibited the same morphology. These observations support our concept of *R. paratrygoni* and suggest that molecular cohesion is not correlated with morphological uniformity – at least for worm size and number of proglottids.

Additional evidence that the large and small specimens identified as *R. paratrygoni* should be assigned to the same species comes from observations of the mating behavior of the worms. In *R. paratrygoni*, a euapolytic cestode, mating occurs between free proglottids subsequent to shedding from the strobila. In this study, free proglottids were frequently observed *en copula* in the stingray spiral intestine, including different-sized proglottids such as mature and gravid proglottids. In these cases, both small (e.g., 15 mm) and large (e.g., 65 mm) mature worms were also present in the spiral intestine. Although it is unknown whether the large and small proglottids observed en copula originated from large and small worms, respectively, this occurrence suggests that large and small *R. paratrygoni* are reproductively compatible, and therefore conspecific.

*Rhinebothrium paratrygoni* most closely resembles *Rhinebothrium copianullum* Reyda, 2008, one of two other species of *Rhinebothrium* reported from South American freshwater stingrays to date. Both species have few testes (i.e., less than 15), are craspedote with 100 s of proglottids (266–1,060 and 456–880), most of which are wider than long. However, the bothridia of *R. paratrygoni* are fusiform in shape, and lack a constriction at their center, whereas those of *R. copianullum* are eliptoid in shape, and are constricted at their center. In addition, the proximal bothridial surfaces of *R. paratrygoni* are evenly covered with acicular filitriches and coniform spinitriches (Fig. 4D), whereas those of *R. copianullum* (see Reyda [27]) possess acicular filitriches and gladiate spinitriches that are restricted to the surfaces that correspond to the distal surface loculi, and only capilliform filitriches on the areas that correspond to the distal surface transverse septa. In addition, the spinitriches on the cirrus are shorter in length in *R. paratrygoni* than they are in *R. copianullum* (2–2.5 vs. 8.3–9.5).

The specific identity of the type host of *R. paratrygoni* remains a mystery. Rego and Dias [41] reported it as *Elipesurus* sp., but *Elipesurus* Jardine, 1843, is considered a *genus inquirendum* according to the recent revision of the Potamotrygonidae by de Carvalho *et al.* [19]. In addition, the specific epithet of *R. paratrygoni* could lead one to believe that it infects species of the freshwater stingray genus *Paratrygon*, but *Paratrygon* is not found in the Paraná River Basin; all freshwater stingray species in the Paraná belong to the genus *Potamotrygon* [19]. The name for the specific epithet is an unfortunate coincidence; the specific epithet *paratrygoni* was assigned at the time by Rego and Dias [41] to denote familial membership of *Elipesurus* in the freshwater stingray family Paratrygonidae Fowler, 1948, but Potamotrygonidae Garman, 1913 is now considered the valid name of the family [19]. Based on current distribution data for freshwater stingrays [19], it is likely that the type host was actually a species of *Potamotrygon*. However, since we encountered *R. paratrygoni* in two of the four *Potamotrygon* species examined from the type locality (i.e., *P. motoro* and *P. falkneri*), either species is an equally likely candidate. We doubt, however, that we will ever have an unambiguous answer for this question.

*Rhinebothrium copianullum* Reyda, 2008. Redescription

Fig. 6

**Figure 6:**
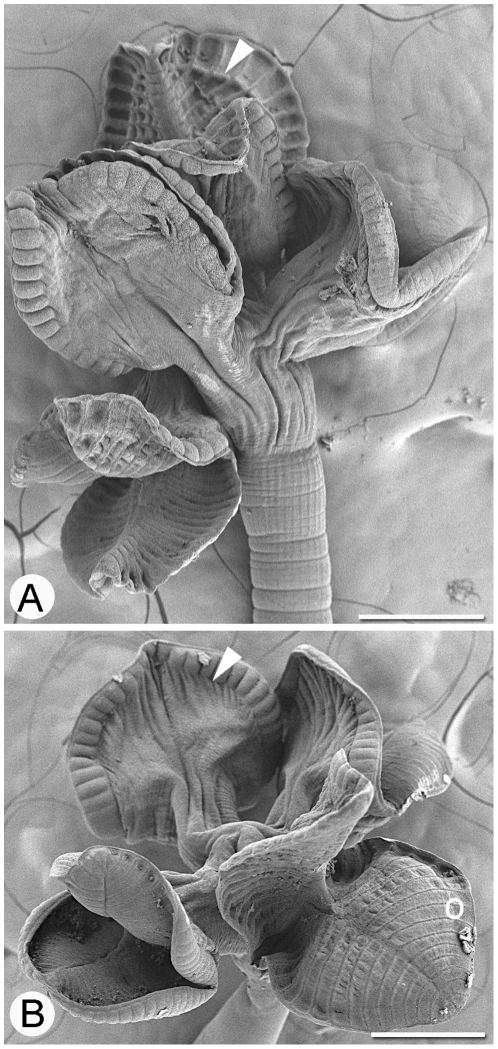
Scoleces of *Rhinebothrium copianullum*. **A**. Scolex in which marginal longitudinal septa are visible, indicated with white arrowhead. B. Scolex in which marginal longitudinal septa are visible on the proximal bothridial surface, indicated by white arrowhead. White circle indicates position of marginal longitudinal septum on distal surface. Scale bar: A–B, 200 µm.

*Redescription* (based on the Holotype, ten paratypes, eight specimens deposited by Reyda [27] as *Rhinebothrium* sp. 1, and newly collected vouchers, consisting of 96 whole mounts of mature worms; including 24 molecular vouchers partially measured, 5 free gravid proglottids, 3 pairs of proglottids en copula, cross sections of 3 strobilae, longitudinal sections of 2 scoleces, and 13 scoleces and 3 proglottids prepared for SEM): Worms euapolytic, craspedote, 10–68 (28±12; n = 91) mm long, greatest width 710–2100 (1116±233; n = 80) at level of scolex; 128–880 (305±140; n = 89) proglottids per worm. Scolex (Fig. 6) consisting of scolex proper bearing 4 stalked bothridia. Bothridia eliptoid-shaped, slightly constricted at center, with muscular rims, 730–1050 (901±98; n = 15) long, 320–750 (503±92; n = 43) wide, divided by 31–43 (36±4; n = 11) transverse septa and 1 medial longitudinal septum into 63–87 (74±8; n = 11) transversely oriented loculi; anterior and posterior halves of each bothridium approximately equal in width. Medial longitudinal septum extending from posterior margin of anteriormost loculus to posterior margin of bothridium. Two additional, marginal longitudinal septa present, but only visible in some bothridia (Fig. 6B). Anteriormost loculus single, 30–62 (45±9; n = 38) long, 32–62 (49±7; n = 45) wide; posteriormost loculi double, 35–55 (44±2; n = 20; *n* = 25) long, 25–45 (33±7; n = 19; *n* = 25) wide. Stalks 81–500 (230±96; n = 61; *n* = 74) long, 70–309 (145±45; n = 65; *n* = 81) wide, attached to bothridia at middle or slightly posterior to middle of bothridium. Cephalic peduncle lacking; neck varying in length.

Proximal surfaces of bothridia covered with acicular filitriches and coniform spinitriches (Fig. 25 in Reyda [27]), except for the edges and narrow bands that correspond to the position of transverse septa on the distal bothridial surfaces (Fig. 26 in Reyda [27]), which only bear only acicular filitriches. Distal surfaces of bothridia with acicular filitriches and coniform spinitriches on surfaces of all septa (including transverse septa, medial longitudinal septum, and marginal longitudinal septa) and on middle portions of loculi, with edges of loculi bearing only acicular filitriches (see Fig. 27 in Reyda [27]). Bothridial rim with acicular filitriches, and a margin of capilliform filitriches (see Fig. 25 in Reyda [27]). Stalks with acicular filitriches and coniform spinitriches (see Fig. 28 in Reyda [27]). Neck and strobila (see Fig. 29 in Reyda [27]) with capilliform filitriches.

Strobila: Greatest proglottid width 290–1200 (473±139; n = 87) near posteriormost proglottids. Majority of proglottids wider than long (Fig. 20); posteriormost 0–32 (8±7; n = 87) proglottids longer than wide; mature proglottids 2–122 (22±24; n = 86) in number, including 0–55 (5±11; n = 81) proglottids in which testes have atrophied and vas deferens are filled with sperm (Fig. 20 in Reyda, 2008). No gravid proglottids observed on strobila.

Terminal proglottids (Fig. 20 in Reyda [27]) 340–1550 (805±252; n = 105) long, 190–650 (349±93; n = 105) wide, length to width ratio 0.8–4.4 (2.1±0.8; n = 44). Genital pores marginal, irregularly alternating, 58–79% (69±5; n = 82) of proglottid length from posterior end. Testes in mature proglottids irregularly oval in dorsal view (Fig. 19 in Reyda [27]), 45–115 (77±16; 87; n = 189) long, 30–105 (56±14; n = 87; *n* = 189) wide, all in primary field, 4–12 (6±1; n = 113; *n* = 241) in total number, 1–2 layers deep, in as many as 4 irregular columns, extending from near anterior margin of proglottid to level of genital pore, rarely extending to anterior margin of ovary on aporal side (Fig. 19 in Reyda [27]). Vas deferens in terminal proglottids coiled, spanning from posterior third of testicular field to near ovarian isthmus, entering cirrus sac at anterior margin. Cirrus sac elongate oval or triangular, bent posteriorly, slender in subterminal mature proglottids (Fig. 19 in Reyda [27]), extending medially to, or well past, midline of proglottid, extending posteriorly to anterior ovarian margin or to ovarian isthmus, containing coiled cirrus. Cirrus sac in terminal proglottids 140–380 (254±51; n = 88) wide, 85–190 (127±24; n = 88) long. Everted cirrus (Fig. 30 in Reyda [27]) 310–500 (378±58; n = 6; *n* = 16) long including expanded base; base 75–130 (94±13; n = 4; *n* = 14) wide, covered with capilliform filitriches and with large coniform (termed “rostrate” in Reyda [27]) spinitriches; coniform spinitriches of cirrus base 8–10.5 (9.5±0.7; n = 11; *n* = 19) long (Fig. 31 in Reyda [27]), distal portion of cirrus 50–75 (65±11; n = 5; *n* = 6) wide, covered with capilliform filitriches and coniform spinitriches (Fig. 32 in Reyda [27]); coniform spinitriches on cirrus distal portion approximately 1.3 long. Vagina thick–walled, sinuous, varying in width along its length, with anterior kink at point where it turns laterally, with conspicuous muscle fibers and darkly staining cells in walls, extending from ootype along medial line of proglottid to anterior margin of cirrus sac, then laterally to common genital atrium. Vaginal sphincter present (Fig. 20 in Reyda [27]). Antero-medial portion of vagina, adjacent to cirrus sac, expanded (Fig. 20 in Reyda [27]). Proximal portion of vagina slightly expanded. Ovary near posterior end of proglottid, lobulated, H-shaped in frontal view, tetra-lobed in cross section (Fig. 22 in Reyda [27]), symmetrical, 130–460 (295±76; n = 79) long, maximum width 120–491 (235±68; n = 75), occupying 27–58% (37±7; 75) of proglottid length; ovarian isthmus located near mid-point of ovary. Anterior margin of ovary 50–320 (145±58; n = 74) short of genital pore. Mehlis' gland posterior to ovarian isthmus. Vitellarium follicular, vitelline follicles 15–50 (29±7; n = 89; *n* = 187) long, 5–42 (20±6; n = 89; *n* = 187) wide, 1 dorsal and 1 ventral column on each side of proglottid, extending from posterior to anterior margin of proglottid, uninterrupted or slightly interrupted ventrally and/or dorsally by cirrus sac and vagina. Uterus ventral, sacciform, extending from posterior margin of ovary to near anterior margin of proglottid.

Free gravid proglottids 1270–2000 (1614±313; n = 7) long, 450–700 (596±81; n = 7) wide; genital pores of gravid proglottids 57–69% (64±0.0; n = 9) of proglottid length from posterior end. Eggs (Fig. 23 in Reyda [27]) embryonated in utero, spherical or semi-spherical, 18–25 (23±3; n = 5) long; oncospheres 17–20 (18±1; n = 5) long, 12–15 (14±1; n = 5) wide; oncospheral hooks 6–8 (7±1; n = 3) long. Proglottids observed en copula of equal or unequal length.

### Taxonomic summary

#### Material examined and material deposited

Holotype, USNPC No. 99943; paratypes, USNPC No. 99944, LRP Nos. 4082–4091, MZUSP NOS. 6392a–6392d, MHNP Nos. 2333–2334. Voucher specimens deposited by Reyda [27] as *Rhinebothrium* sp. 1 (LRP Nos. 4092–4099; 4113–4114; 4145–4146) Voucher specimens of *R. copianullum* deposited as part of the current study: Seventy-two whole mounts (MZUSP 6057a, 6057d, 6058a–b, 6059a–b, 6060, 6061a–b, 6062a, 6063a, 6064a, 6065, 6066a, 6067a, 6068a, 6069a, 6070a–b, 6071, 6072a–b, 6073a, 6074a, 6075a–b, 6076a, 6077a, 6078a, 6079a–b, 6080, 6081a, 6082a–b, 6083a–b, 6084a–c, 6085a–b, 6086a, 6097b, 6099c; LRP 7638–7651; USNPC 104702, 104703 (2 slides), 104704, 104705 (2 slides), 104706–104710, 104711 (2 slides)), five free gravid proglottids (MZUSP 6054a–b; LRP 7635, 7636; USNPC 104700), three pairs of proglottids en copula (MZUSP 6055a; LRP 7637; USNPC 104701), twenty scoleces prepared for SEM (MZUSP 6015–6024, 6026–6028, 6033–6037, 6043), and three proglottids from a single worm prepared for SEM (MZUSP 6025), cross sections of strobilae of three worms (MZUSP 5982–5985, 5986–5990, 5996–6005), longitudinal sections of two scoleces (MZUSP 5991–5993, 5994–5995), and 27 voucher specimens (i.e., hologenophores) of sequenced worms (MZUSP 5958–5981, 6962–6964 for GenBank Nos. JF803694–JF803718, JF803726–JF803728).

#### Type host

*Paratrygon aiereba* (Müller and Henle, 1841), Discus ray.

#### Additional hosts

*Potamotrygon leopoldi*, *Potamotrygon henlei*, *Potamotrygon* sp. (tap1), *Potamotrygon* sp. (tap2), *Potamotrygon* sp. (toc2), *Potamotrygon* sp. (cururu), *Potamotrygon orbignyi*.

#### Accidental hosts

Immature specimens encountered in *Potamotrygon motoro*, *Potamotrygon tatianae*, and *Potamotrygon schroederi*.

#### Type locality

Madre de Dios River at Boca Manu, Madre de Dios Department, Peru (12°17′047″S, 70°53′086″W).

#### Additional localities

Rio Negro, Negro sub-basin, Amazon Basin, near Barcelos, Amazonas State, Brazil (Lat: 0°58′48″S Long: 62°55′12″W); Rio Negro/Paraná Zamula, Negro sub-basin, Amazon Basin, near Barcelos, Amazonas State, Brazil (Lat: 0°51′58″S Long: 62°46′34″W); Rio Negro/Mouth of River Demeri, Negro sub-basin, Amazon Basin, near Barcelos, Amazonas State, Brazil (Lat: 0°46′12″S Long: 62°56′24″W); Rio Tarauacá, Juruá sub-basin, Amazon Basin, at Tarauacá, Acre State, Brazil (Lat: 8°4′6″S Long: 70°43′5″W); Rio Yavari, Solimões-Yavari-Itacuaí sub-basin, Amazon Basin, at Benjamin Constant, Amazonas State, Brazil (Lat: 4°19′51″S Long: 70°4′31″W); Teles Pires River, Tapajós sub-basin, Amazon Basin, at Alta Floresta, Mato Grosso State, Brazil (Lat: 8°52′48″S Long: 57°22′48″W); Xingú River, Xingú sub-basin, Amazon Basin, at São Paulo do Xingú, Pará State, Brazil (Lat: 6°39′36″S Long: 52°0′0″W); Tocantins River, Tocantins-Araguaia sub-basin, Amazon Basin, at Marabá, Pará State, Brazil (Lat: 5°21′36″S Long: 49°7′48″W); Paraná River, Tocantins-Araguaia sub-basin, Amazon Basin, at Paranã, Tocantins State, Brazil (Lat: 12°15′S Long: 47°48′W); Manuel Alves River, Tocantins-Araguaia sub-basin, Amazon Basin, at Ipueiras, Tocantins State, Brazil (Lat: 11°18′36″S Long: 48°27′36″W); Araguaia River, Tocantins-Araguaia sub-basin, Amazon Basin, at São Miguel do Araguaia, Goiás State, Brazil (Lat: 12°56′24″S Long: 50°31′12″W); Araguaia River, Tocantins-Araguaia sub-basin, Amazon Basin, at Caseara, Tocantins State, Brazil (Lat: 9°16′12″S Long: 49°57′36″W); Igarapé Cururu River, Amazon Basin, at Cachoeira do Arari, Ilha de Marajó, Para State, Brazil (Lat: 1°0′36″S Long: 48°57′36″W).

#### Site of infection

spiral intestine.

#### Additional vouchers deposited

Cross sections of 3 strobilae (MZUSP 6006–6011, 6012–6014, 6050–6053) and 121 whole mounts (MZUSP 6029–6032, 6038–6049, 6057e–k, 6059g, 6062b–c, 6063b–d, 6066b–d, 6067b–d, 6068b, 6069d, 6070f–h, 6072g, 6073b–e, 6074b–d, 6075e–f, 6076b–d, 6077b–d, 6084h, 6085c, 6086b, 6087a–b, 6088a–c, 6089–6091, 6092a–b, 6093, 6094a–c, 6095a–c, 6096–6098, 6099a–b, 6100, 6101a–b, 6102a–b, 6103a–c, 6104a–e, 6105, 6106a–b, 6107–6109, 6110a–l, 6111, 6112a–d, 6113a–c, 6114).

### Remarks

*Rhinebothrium copianullum* Reyda, 2008 was described based on whole mounts of 10 worms, 2 free gravid proglottids, 2 egg mounts, cross sections of 3 proglottids, longitudinal sections of 1 scolex, and 2 scoleces and 5 proglottids prepared for SEM, collected from the spiral intestines of four *Paratrygon aiereba* in the upper Amazon Basin in southeastern Peru (Reyda [27]). Our redescription included re-examination of the type series, and newly collected vouchers consisting of 106 whole mounts – including 24 molecular vouchers partially measured, as well as numerous free gravid proglottids, proglottids *en copula*, and specimens examined histologically, or with SEM. The striking differences between these two samples are biogeographical and host representations. The material available to Reyda [27], and used to define his concept of *R. copianullum*, was limited in host and geographic representation. The material available here, by contrast, consisted of mature specimens collected from eight stingray species (see Table S1) and numerous major rivers throughout the Amazon Basin (see Fig. 1). Reyda [27] also collected a putative new species of *Rhinebothrium* (referred as sp. 1) from 7 specimens of *Potamotrygon motoro* and 14 specimens of *Potamotrygon tatianae* from the same locality. Those *Rhinebothrium* specimens are here considered *R. copianullum* and are incorporated into the revision. Thus, as part of this redescription of *R. copianullum*, we have expanded its distribution to include many more host species and localities throughout the Amazon Basin.

The broader biogeographic and host representation of the redescription of *R. copianullum* is reflected in the higher variability reported for *R. copianullum*. The following examples illustrate how the lower limits of the ranges of several morphometric and meristic attributes of *R. copianullum* have been expanded. For instance, *R. copianullum* was reported initially as being 30–68 mm long, but is shown here to have 10–68 mm in length. The number of proglottids per worm has expanded from 456–880 to 128–880, number of transverse septa from 39–43 to 31–43, and total number of testes per proglottid from 6–12 to 4–12.

The broad amount of size variation observed in the revised concept of *R. copianullum* is more than is typically characterized for other rhinebothriine cestode species (see Remarks for *R. paratrygoni*). It would seem intuitive to recognize mature specimens that correspond to the minimum, median, and maximum total lengths reported here for *R. copianullum* as different species of *Rhinebothrium*. In fact, the attempt to identify a morphological feature that would enable subdivision of the specimens of *R. copianullum* into different species was a major focus of Reyda's dissertation [51]. That study included light microscope examination of whole mounts of mature worms and free proglottids, strobila cross-sections and scolex longitudinal sections; as well as scanning electron microscope examination of scoleces and proglottids. Because these efforts did not reveal any morphological evidence to split *R. copianullum*, additional (molecular) data were explored.

Sequences representing the 28 specimens that were assigned to *R. copianullum* formed a monophyletic group (Clade D, Fig. 2), with a bootstrap support value of 73. Clade D (Fig. 2) consists of haplotypes from specimens from four stingray species from most of the rivers sampled in the Amazon Basin (Fig. 1). This clade encompasses higher nucleotide diversity (π = 0.06796), and, hence, wider uncorrected pairwise patristic distances variation (ranging from 0 to 0.14442) than the *R. paratrygoni* clade (See Clade A, Fig. 2). However, the internal structure of Clade D does not appear to be correlated to any characteristic that could be used to recognize additional putative species within nested clades. The most obvious attribute would be total length, for which no pattern emerges when it is mapped onto the terminals in Clade D for which this datum was available (Fig. 2). In addition, haplotypes of specimens from a single river did not completely group together (Fig. 2). Whether mature specimens were short, medium, or long, all individuals attributed to *R. copianullum* possess eliptoid-shaped bothridia with a single median, and two lateral, longitudinal septa with complex microthrix distribution patterns (as detailed above), and a slight constriction at the center; a craspedote strobila consisting of 100 s of proglottids, most of which are wider than long; mature proglottids that possess a vagina with a well-pronounced sphincter and anterior kink; and a cirrus sac containing a cirrus with large coniform spinitriches. These attributes provided that morphological cohesion for our concept of *R. copianullum*, and suggest that worm length and/or numbers of proglottids are meaningless to distinguish lineages within this group.

Additional evidence that the large and small specimens identified as *R. copianullum* are conspecific comes from observations of free proglottids *en copula*, as for *R. paratrygoni*, in which pairs of free proglottids of different sizes were observed en copula. Mature *R. copianullum* worms of different sizes were often observed in the same individual stingray spiral intestine in which free proglottids were found *en copula*, again suggesting that there is reproductive compatibility between large and small specimens.

The additional sampling and specimens also greatly change the view of host specificity of *R. copianullum*. Reyda [27] considered *R. copianullum* to be highly host specific (oioxenous), parasitizing only *P. aiereba*. However, he acknowledged that “ a full understanding of the host specificity of *Rhinebothrium* in potamotrygonids requires further taxonomic study of specimens in multiple host species and localities” (Reyda [27]: 696–697). In fact, among freshwater lineages of *Rhinebothrium*, *R. copianullum* seems to have the lowest level of host specificity; as a result of our work mature specimens have been reported form seven species of *Potamotrygon*, and immature specimens from three other *Potamotrygon* species.

Marginal longitudinal septa were visible in a subset of the *R. copianullum* specimens. The two scoleces shown in Fig. 6 include one in which marginal longitudinal septa are evident on the distal bothridial surface (white arrow, Fig. 6a), and one in which evidence of marginal longitudinal septa can only be seen on the backs (i.e., proximal surface) of the bothridia (white arrow, Fig. 6b). Even in scoleces in which marginal longitudinal septa are not visible, however, the underlying septa or muscle bundles correspond to areas that bear both acicular filitriches and coniform spinitriches (white circle, Fig. 6b). The microthrix data suggest that all *R. copianullum* specimens possess marginal longitudinal septa, but that their visibility varies, probably due to the state of muscle contraction at the time of specimen fixation. The portions of facial loculi lateral to marginal longitudinal septa were not considered “marginal loculi” in other rhinebothriine genera like *Anthocephalum*, and were not counted in addition to the transversely oriented loculi.

*Rhinebothrium copianullum* is most similar to *R. paratrygoni*, the only other species of *Rhinebothrium* reported from South American freshwater stingrays that is considered valid here. Both species are craspedote with 100's of proglottids (266–1,060 and 128–880), most of which are wider than long. However, whereas in *R. copianullum* the bothridia are eliptoid in shape, with a slight constriction at their center, those of *R. paratrygoni* are fusiform (or diamond) -shaped, lacking a central constriction. In addition, the cirrus of *R. copianullum* is larger relative to the proglottid than in *R. paratrygoni*. The two species also differ in microthrix patterns. Whereas the proximal bothridial surfaces of *R. paratrygoni* are evenly covered with acicular filitriches and coniform spinitriches (Fig. 4D), the proximal bothridial surfaces of *R. copianullum* possesses acicular filitriches and coniform spinitriches on areas that correspond to the loculi of the distal surface, and only capilliform filitriches on areas that correspond to the underlying transverse septa. In addition, the coniform spinitriches on the cirrus are smaller in *R. paratrygoni* than in *R. copianullum* (2–2.5 vs. 8.3–9.5).

*Rhinebothrium brooksi* sp. n. Description.

urn:lsid:zoobank.org:act:CA391692-D2DA-444A-804B-A850A0E2F9E2

Figs. 7, 8, 9

**Figure 7:**
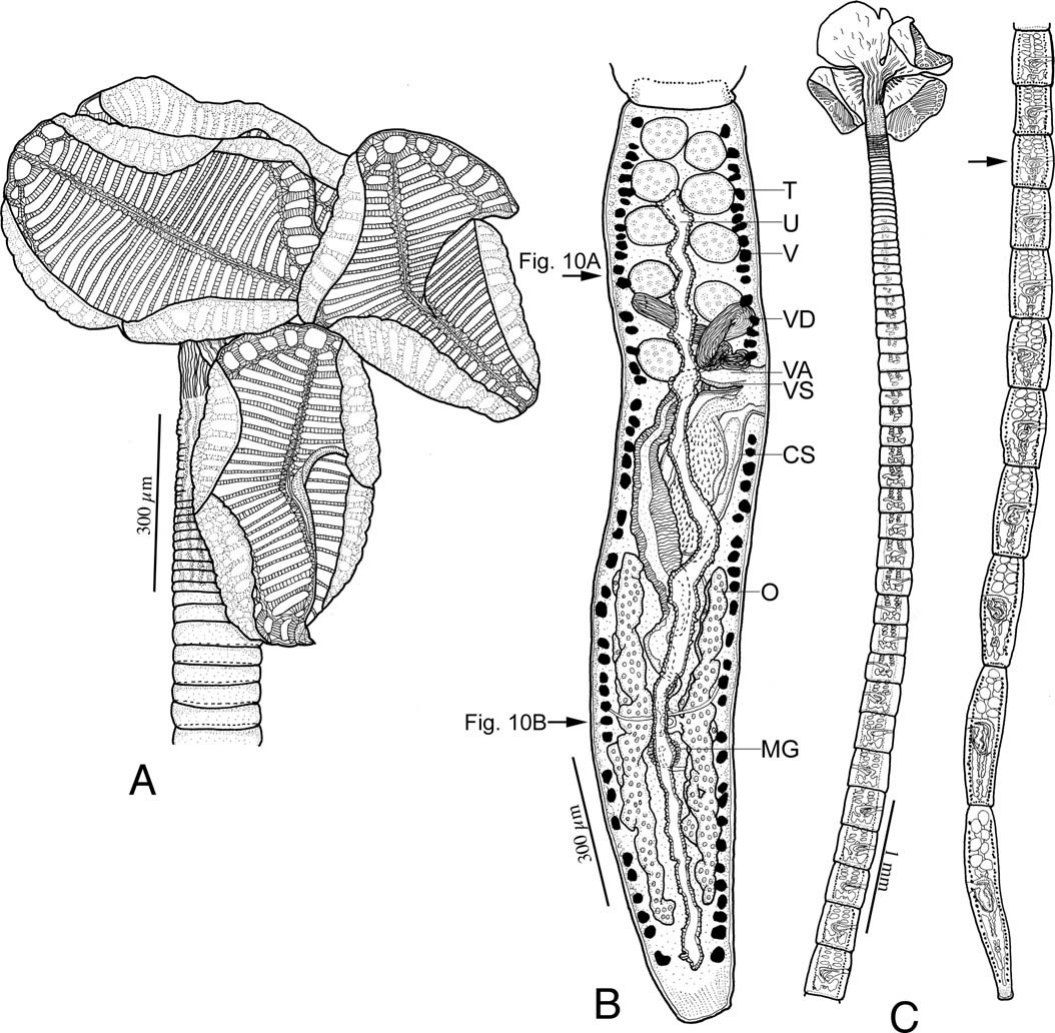
Line drawings of *Rhinebothrium brooksi* n. sp. A. Scolex of Holotype (MZUSP 6124). B. Terminal mature proglottid of Paratype (USNPC 104712). Arrows indicate locations of sections shown in Figure. 10. C. Anterior and posterior portions of whole worm (Paratype, MZUSP 6123). Arrow indicates anterior most mature proglottid. *Abbreviations*: CS, Cirrus sac; MG, Mehlis' gland; O, Ovary; T, Testes; U, Uterus; V, Vitellaria; VA Vagina; VD, vas deferens; VS Vaginal sphincter.

**Figure 8:**
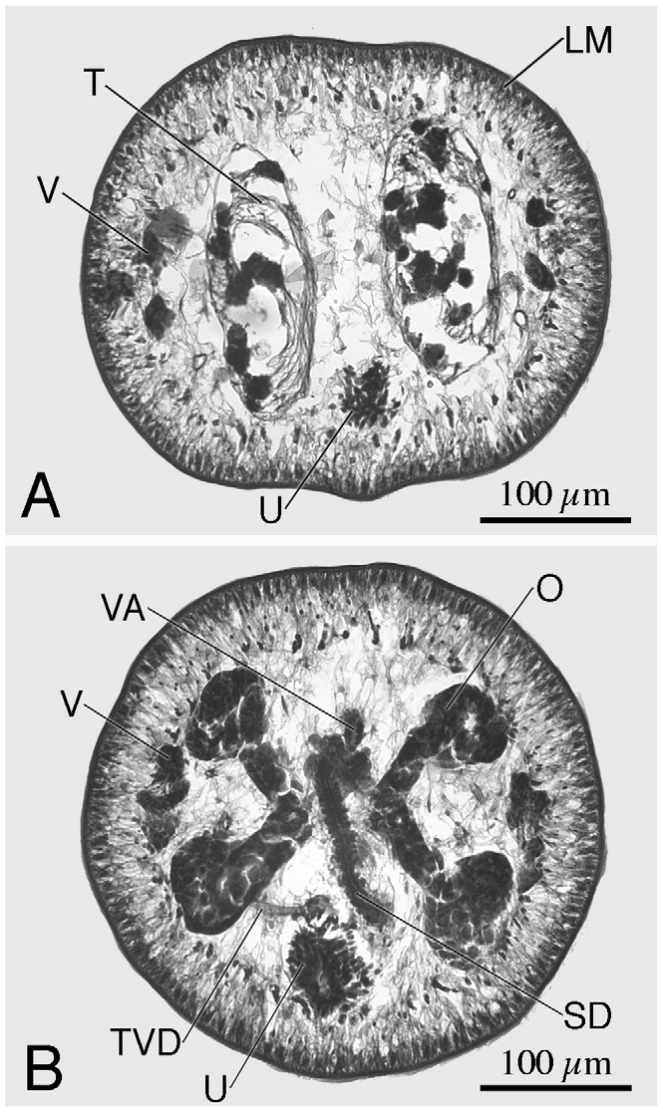
Cross-section through mature proglottid of *Rhinebothrium brooksi* n. sp. A. Section at level of testes. B. Section at level of ovarian isthmus. *Abbreviations:* LM, Longitudinal muscles; O, Ovary; SD Sperm duct; T, Testes; TVD, Transverse vitelline duct; U, Uterus; V, Vitellaria; VA Vagina.

**Figure 9:**
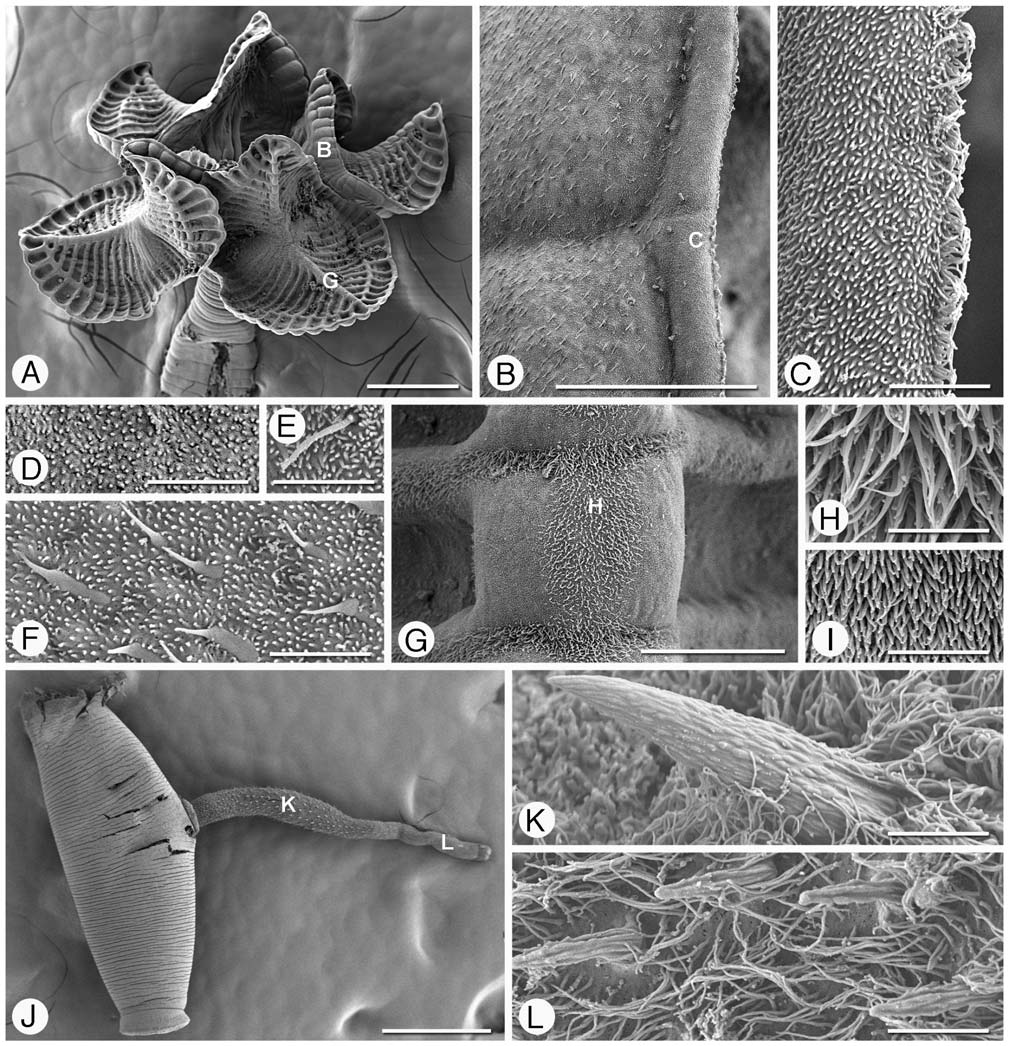
Scanning electron micrographs of *Rhinebothrium brooksi* n. sp. Scolex, Figures A–I. Small letters indicate locations of details shown in B and G. A. Scolex. B. Proximal surface of rim of bothridium. Small letter indicates location of C. C. Proximal bothridial surface near bothridial rim. D. Proximal surface near anterior of bothridium. E. Cilium on proximal bothridial surface. F. Proximal surface near middle of bothridium. G. Transverse septum on distal bothridial surface. Small letter indicates location of H. H. Longitudinal septum. I. Stalk surface. Cirrus, Figures J–L. J. Free proglottid with everted cirrus. Small letters indicate location of K and L. K. Coniform spinithrix and capilliform filitriches on cirrus base. L. Coniform spinitriches and capilliform filitriches on distal portion of cirrus. Scale bars: A, 200 µm; B, 20 µm; C–F, 2 µm; G, 20 µm; H–I, 2 µm; J, 200 µm; K–L, 2 µm.

### Description

*Diagnosis* (based on whole mounts of 39 mature worms; including 6 molecular vouchers partially measured, cross sections of 3 strobilae, longitudinal sections of 5 scoleces, and 10 scoleces and 1 proglottid prepared for SEM): Worms (Fig. 7C) euapolytic, slightly craspedote, 6–27 (16±5; n = 33) mm long, greatest width 510–1,500 (924±213; n = 28) at level of scolex; 53–139 (83±21; n = 33) proglottids per worm. Scolex (Figs. 7A, 9A) consisting of scolex proper bearing 4 stalked bothridia. Bothridia eliptoid-shaped, slightly constricted at center, with muscular rims (Fig. 9B), 540–640 (582±38; n = 4; *n* = 5) long, 390–800 (570±129; n = 22) wide, divided by 27–32 (29±2; n = 10; *n* = 15) transverse septa and 1 medial longitudinal septum into 55–65 (59±3; n = 10; *n* = 15) transversely oriented loculi; anterior and posterior halves of each bothridium approximately equal in width. Medial longitudinal septum extending from posterior margin of anteriormost loculus to posterior margin of bothridium. Two additional, marginal longitudinal septa present (Figs. 7A, 9A). Anteriormost loculus single, 32–55 (44±6; n = 19; *n* = 20) long, 37–72 (54±8; n = 19; *n* = 20) wide; posteriormost loculi double, 32–65 (48±8; n = 12) long, 27–67 (41±11; n = 13; *n* = 14) wide. Stalks 110–260 (170±47; n = 13; *n* = 14) long, 90–150 (126±22; n = 13; *n* = 14) wide, attached to bothridia at middle or slightly posterior to middle of bothridium. Cephalic peduncle lacking; neck varying in length.

Entire proximal surface of bothridia covered with acicular filitriches (Fig. 9D), proximal bothridial surface adjacent to middle of bothridia with acicular filitriches and coniform spinitriches (Fig. 9F), and with a few cilia (Fig. 9E). Distal surfaces of bothridia with acicular filitriches and coniform spinitriches on surfaces of all septa, and on middle portions of loculi, with edges of loculi bearing only acicular filitriches (Figs. 9G, 9H). Bothridial rim (Fig. 9B) with acicular filitriches, and a margin of capilliform filitriches (Fig. 9C). Stalks (Fig. 9I), neck and strobila with capilliform filitriches.

Strobila: Greatest proglottid width 220–500 (331±67; n = 29) at or near posteriormost proglottids. Majority of proglottids wider than long; posteriormost 6–34 (16±8; n = 28) proglottids longer than wide (Fig. 7C); mature proglottids 4–13 (8±2; n = 29) in number, including 0–2 (0.3±0.6; n = 29) proglottids in which testes have atrophied and vas deferens are filled with sperm. No gravid proglottids observed on strobila.

Terminal proglottid (Fig. 8B) 600–2,150 (1,232±316; n = 33) long, 200–450 (281±58; n = 33) wide, length to width ratio 1.4–2.4 (4.6±1.4; n = 30). Genital pores marginal, irregularly alternating, 57–73% (68±3.9; n = 28) of proglottid length from posterior end. Testes in mature proglottids irregularly oval in dorsal view (Fig. 7B), 65–120 (93±13; n = 29; *n* = 58) long, 40–105 (70±13; n = 29; *n* = 58) wide, all in primary field, 7–13 (9±2; n = 36; *n* = 57) in total number, 1 layer deep (Fig. 8A), in 2 irregular columns, extending from near anterior margin of proglottid to level of genital pore, rarely extending to anterior margin of ovary on poral side. Vas deferens in terminal proglottids coiled, spanning from posterior third of testicular field to near ovarian isthmus, entering cirrus sac at anterior margin. Cirrus sac elongate oval or triangular, bent posteriorly, slender in subterminal mature proglottids (Fig. 7C), extending medially to, or well past, midline of proglottid, extending posteriorly to anterior ovarian margin or to near ovarian isthmus, containing coiled cirrus. Cirrus sac in terminal proglottids 210–370 (286±44; n = 30) wide, 90–170 (126±n = 18; *n* = 30) long. Everted cirrus (Fig. 9M) 320–570 (445±177; n = 2) long, ∼70 (69±2; n = 2) wide. Proximal portion of cirrus covered with capilliform filitriches and with large coniform spinitriches; coniform spinitriches of proximal portion of cirrus 7.6–8 (7.8±0.3; n = 1; *n* = 2) long, 2.1–2.4 (2.2±0.2; n = 1; *n* = 2) wide (Fig. 9K), coniform spinitriches of distal portion of cirrus 3–4 (3.5±0.7; n = 1; *n* = 2) long, 1 (1±0; n = 1; *n* = 2) wide (Fig. 9L). Vagina thick-walled, sinuous, varying in width along its length, with conspicuous muscle fibers and darkly staining cells in walls (Fig. 7B), extending from ootype along medial line of proglottid to anterior margin of cirrus sac, then laterally to common genital atrium; middle portion of vagina thick walled. Vaginal sphincter present (Fig. 7B). Antero medial portion of the vagina, adjacent to cirrus sac, greatly expanded (Fig. 7B). Proximal portion of vagina slightly expanded. Ovary near posterior end of proglottid, lobulated, H-shaped in frontal view, tetra-lobed in cross section (Fig. 8B), symmetrical, 250–790 (492±135; n = 31) long, maximum width 115–270 (200±42; n = 26), occupying 32–50% (40±6; n = 28) of proglottid length; ovarian isthmus located near or slightly anterior to mid-point of ovary. Anterior margin of ovary 100–370 (220±70; n = 27) short of genital pore. Mehlis' gland posterior to ovarian isthmus. Vitellarium follicular, vitelline follicles 15–42 (28±6; n = 29; *n* = 58) long, 10–30 (18±5; n = 29; *n* = 58) wide, 1 dorsal and 1 ventral column on each side of proglottid, extending from posterior to anterior margin of proglottid, uninterrupted or slightly interrupted ventrally and/or dorsally by cirrus sac and vagina. Uterus ventral, sacciform, extending from posterior margin of ovary to near anterior margin of proglottid.

Free gravid proglottids and eggs not observed.

### Taxonomic summary

#### Type host

*Paratrygon aiereba* (Müller & Henle, 1841), Discus ray.

#### Additional hosts

*Potamotrygon orbignyi*.

#### Type locality

Rio Negro, Negro sub-basin, Amazon Basin, near Barcelos, Amazonas State, Brazil (Lat: 0°58′48″S Long: 62°55′12″W).

#### Additional localities

Rio Negro/Paraná Zamula, Negro sub-basin, Amazon Basin, near Barcelos, Amazonas State, Brazil (Lat: 0°51′58″S Long: 62°46′34″W); Rio Negro/Mouth of River Demeri, Negro sub-basin, Amazon Basin, near Barcelos, Amazonas State, Brazil (Lat: 0°46′12″S Long: 62°56′24″W); Xingú River, Xingú sub-basin, Amazon Basin, at São Paulo do Xingú, Pará State, Brazil (Lat: 6°39′36″S Long: 52°0′0″W); Tapajós River, Tapajós sub-basin, Amazon basin, at Santarém, Para State, Brazil (Lat: 2°16′48″S Long: 55°0′0″W); Pimental River, Tapajós sub-basin, Amazon basin, at Pimental, Para State, Brazil (Lat: 4°33′36″S Long: 56°15′36″W).

#### Site of infection

Spiral intestine.

#### Holotype

MZUSP 6124 (1 whole mount).

#### Paratypes

Thirty-two whole mounts (MZUSP 6115a–b, 6116a–b, 6117a, 6118–6120, 6121a–b, 6122–6123, 6125a–b, 6126, 6127a, 6128a–c, 6129, 6130a–b; LRP 7652–7656; USNPC 104712 (2 slides), 104713–104715), ten scoleces and one proglottid prepared for SEM (MZUSP 6131–6140 and 6141, respectively), cross sections of strobilae of three worms (MZUSP 6150a–j, 6155a–i, 6156a–f), longitudinal sections of five scoleces (MZUSP 6151a–b, 6152a–c, 6153a–d, 6154a–d, 6159a–b), and six voucher specimens (i.e., hologenophores) of sequenced worms (MZUSP 6142–6147 for GenBank Nos. JF803719–JF803724).

#### Vouchers deposited

Cross sections of one strobila (MZUSP 6148a–f), longitudinal sections of three scoleces (MZUSP 6149a–c, 6157a–b, 6158a–c), and 116 whole mounts (MZUSP 6161a–o, 6162 a–i, 6163a–c, 6164, 6165a–c, 6166–6169, 6170a–c, 6171a–k, 6172, 6173a–c, 6174, 6175a–b, 6176a–p, 6177a–g, 6178a–c, 6179a–h, 6180a–b, 6181a–i, 6182, 6183a–b, 6184, 6185a–b, 6186).

#### Etymology

This species is named in honor of Dan Brooks for his pioneering work on the parasites of potamotrygonids.

### Remarks

*Rhinebothrium brooksi* n. sp. can be distinguished from all 41 described species of *Rhinebothrium*, except *R. copianullum*, with which it overlaps in both geography and host species, in its possession of marginal longitudinal septa on either side of the bothridia. This feature is denoted by a microthrix pattern different than is seen within the loculi, acicular filitriches in combination with coniform spinitriches. *Rhinebothrium brooksi* n. sp. and *R. copianullum* can be distinguished based on microthrix patterns, as well as with features of the strobila. Although both species possess acicular filitriches and coniform spinitriches on their proximal bothridial surfaces, in *R. brooksi* the coniform spinitriches are restricted to the middle portion of the proximal bothridial surface, whereas in *R. copianullum*, the coniform spinitriches are distributed throughout all regions of the proximal bothridial surface that correspond to loculi on the distal surface. *Rhinebothrium brooksi* n. sp. generally possesses fewer proglottids than *R. copianullum* (53–139 vs. 128–880). In *R. brooksi* n. sp., the first square proglottid occurs within the anterior third of the strobila (Fig. 7C), whereas in *R. copianullum* the first square proglottid occurs in the posterior half of the strobila. This feature can also be used to distinguish *R. brooksi* n. sp. from *R. paratrygoni*, the other species of *Rhinebothrium* described from South American potamotrygonids to date; in *R. paratrygoni* the strobila consists of many proglottids that are wider than long, and the first proglottid that is as long as wide occurs posteriorly. *Rhinebothrium brooksi* n. sp. also possesses larger coniform spinitriches on its cirrus than does *R. paratrygoni* (7.6–8 vs. 2–2.5).

In having marginal longitudinal septa, the bothridia of *R. brooksi* n. sp. and *R. copianullum* actually more closely resemble bothridia of species of *Rhinebothroides* Mayes, Brooks, and Thorson, 1981, also from South American potamotrygonids. Morphological studies of *Rhinebothroides* [47], [49] have shown that visibility of marginal longitudinal septa varies among specimens. However, the proglottid morphology of *Rhinebothroides* is completely different than that of *R. brooksi* in that its proglottids have distinctly asymmetrical ovaries, genital pores in the posterior portion of the proglottid, and 20 or more testes [47], [49].

Specimens identified as *R. brooksi* n. sp. were found to nest in a single clade (Clade C, Fig. 2), which is mainly represented by specimens collected in Rio Negro, but we were able to include a single specimen from the Xingú River (Fig. 2). Clade C encompasses a relatively high nucleotide diversity (π = 0.07614) and moderate uncorrected pairwise patristic distances variation (ranging from 0.00175 to 0.11228) in comparison to the other clades of freshwater lineages of Rhinebothrium. Although within this clade the largest worm collected in Xingú River nested basal to the remaining haplotypes from Rio Negro, which in turn exhibited smaller size for those we had total length recorded (see Fig. 2, Clade C), we predict that there is no correlation between cladistic structure and worm size – as we observed for the clades above. The values for total length of the 33 specimens of *R. brooksi* that were measured ranged from 6–27 mm, and despite the morphological variation in total length – hence number of proglottids –, all of the specimens that were examined can be recognized as *R. brooksi* based on their possession of a cirrus with large coniform spinitriches, in combination with the distribution of microtriches on the proximal bothridial surface and a strobila in which the first square proglottid occurs anteriorly.

The cladistic pattern observed in Clade C (Fig. 2) and the close relationship between a haplotype from Xingú River and those from Rio Negro might be explained by the phylogeography of the host. Fehlauer-Ale [75] provided a preliminary phylogeographic study of *Paratrygon aiereba* based on 3 mtDNA genes and suggested that the population in the Xingú River was sister to a large clade of haplotypes of *Paratrygon aiereba* from Rio Negro and Tapajós Rivers, among many other populations. The position of the haplotype of *R. brooksi* n. sp. from the Xingú River relative to those from the Rio Negro parallels the phylogeographic pattern found by Fehlauer-Ale [75] for their hosts. However, a better biogeographical representation of haplotypes of *R. brooksi* n. sp., especially from localities that were not sampled here (i.e., Tapajós River), is further required to explore relationships between *R. brooksi* n. sp. and its hosts.

*Rhinebothrium fulbrighti* sp. n. Description.

urn:lsid:zoobank.org:act:863B1779-A1FD-4D45-AF74-DD2A7CC87962

Figs. 10–11

**Figure 10:**
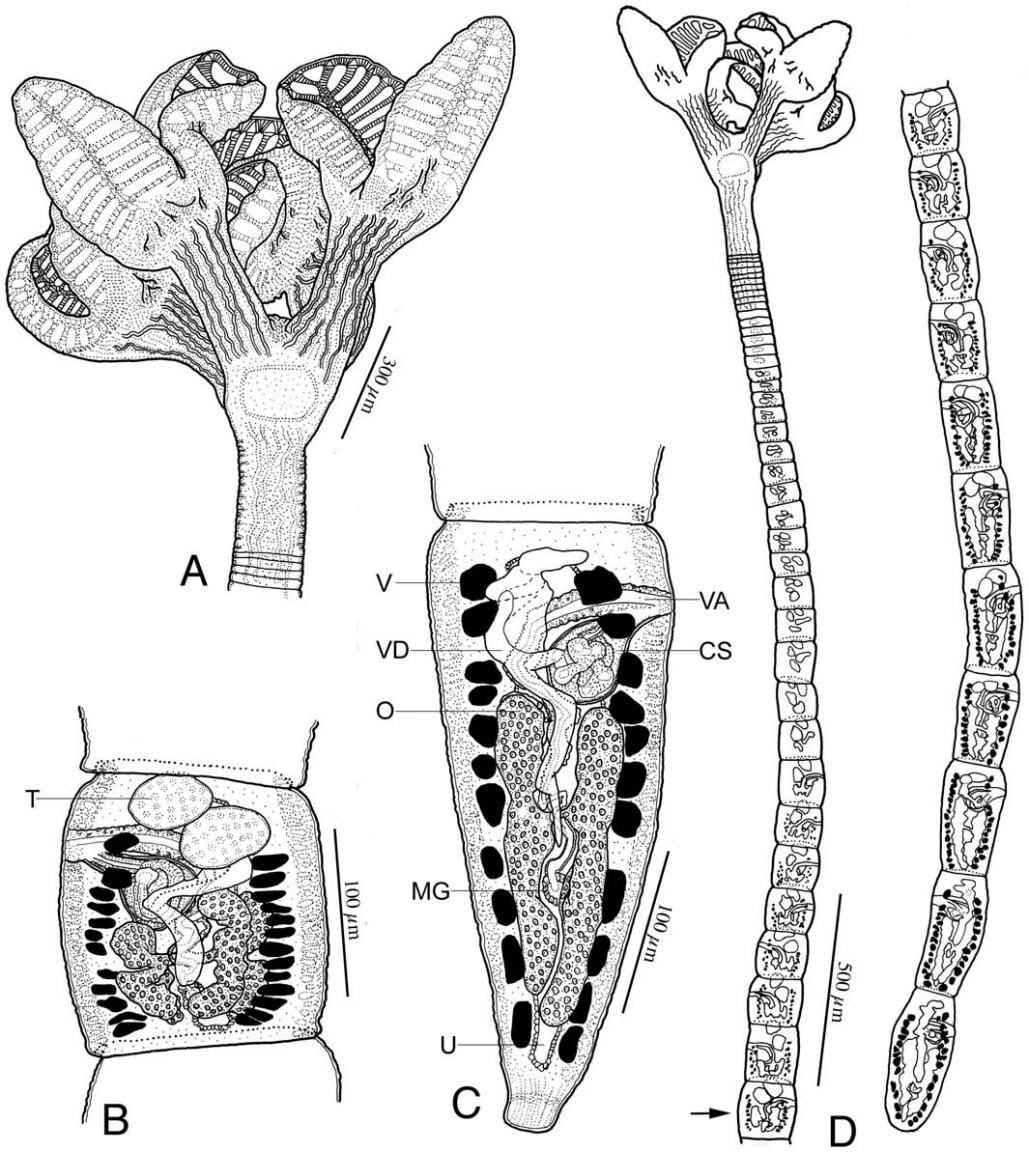
Line drawings of *Rhinebothrium fulbrighti* n. sp. A. Scolex of Holotype (MZUSP 6309c). B. Subterminal mature proglottid of Paratype (USNPC 104730). C. Terminal mature proglottid of Paratype (USNPC 104730). D. Anterior and posterior portions of whole worm (Holotype, MZUSP 6309c). Arrow indicates anterior most mature proglottid. *Abbreviations*: CS, Cirrus sac; MG, Mehlis' gland; O, Ovary; T, Testes; U, Uterus; V, Vitellaria; VA Vagina; VD, vas deferens.

**Figure 11:**
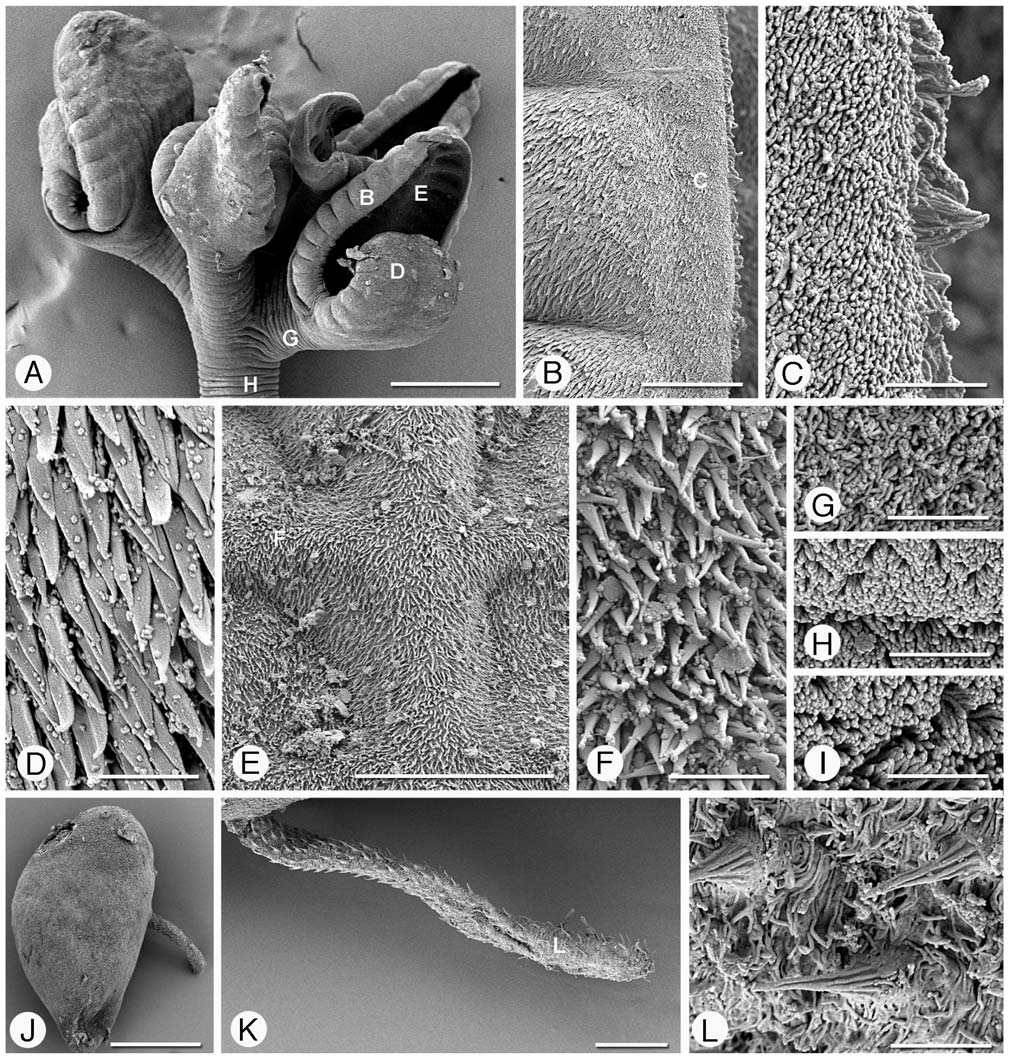
Scanning electron micrographs of *Rhinebothrium fulbrighti* n. sp. Scolex, Figures A–I. Scolex. A. Scolex. Small letters indicate locations of details shown in B, D–E, G–H. B. Proximal surface of rim of bothridium. Small letter indicates location of details shown in C. C. Proximal bothridial surface near bothridial rim. D. Proximal bothridial surface. E. Transverse septum on distal bothridial surface. Small letter indicates location of detail shown in F. F. Transverse septum on distal bothridial surface. G. Stalk surface. H. Cephalic peduncle. I. Strobila surface. Cirrus, Figures J–L. J. Free proglottid with everted cirrus. K. Everted cirrus. Small letter indicates location of detail shown in L. L. Coniform spinitriches with ridges and filitriches on cirrus. Scale bars: A, 100 µm; B, 10 µm; C–D, 2 µm; E, 20 µm; F–I, 2 µm; J, 100 µm; K, 20 µm; L, 2 µm.

### Description

*Diagnosis* (based on whole mounts of 57 mature worms, including 7 molecular vouchers partially measured; 4 free gravid proglottids, cross sections of 4 strobilae, longitudinal sections of 2 scoleces, and 12 scoleces and 16 free proglottids prepared for SEM): Worms (Fig. 10D) euapolytic, craspedote, 3.1–18 (6.3±2.4; n = 57) mm long, greatest width 320–1110 (717±175; n = 49) at level of scolex; 40–168 (66±22; n = 50) proglottids per worm. Scolex (Figs. 10A, 11A) consisting of scolex proper bearing 4 stalked bothridia. Bothridia (Figs. 11B, 11C) eliptoid-shaped, slightly constricted at center, with muscular rims 310–650 (485±87; n = 16; *n* = 17) long, maximum width 220–420 (298±49; n = 24) at anterior half of bothridium, divided by 21–26 (23±1.3; n = 18) transverse septa and one longitudinal septum into 43–53 (47±2.6; n = 18) transversely oriented loculi. Medial longitudinal septum extending from posterior margin of anteriormost loculus to posterior margin of bothridium. Anteriormost loculus single, 25–48 (34±6; n = 37; *n* = 47) long, 32–63 (43±7; n = 40; *n* = 50) wide; posteriormost loculi double 22–50 (33±7; n = 24; *n* = 34) long, 20–42 (30±6; n = 23; *n* = 34) wide. No marginal longitudinal septa observed. Stalks 75–300 (149±58; n = 48; *n* = 60) long, 52–125 (88±19; n = 49; *n* = 60) wide, attached to middle of bothridium. Cephalic peduncle lacking. Neck varying in length.

Proximal surfaces of bothridia covered with acicular filitriches and gladiate spinitriches throughout (Figs. 11B, 11D). Distal surfaces of bothridia covered with acicular filitriches and coniform spinitriches throughout, including surfaces of longitudinal and transverse septa (Figs. 11E, 11F). Bothridial rim with acicular filitriches and a margin of capilliform filitriches (Fig. 11C). Stalks (Fig. 11G), neck (Fig. 11H) and strobila (Fig. 11I) with capilliform filitriches.

Strobila: Greatest proglottid width 90–250 (163±35; n = 49) at or near posteriormost proglottids. Numerous proglottids wider than long; posteriormost 1–36 (11±7; n = 50) proglottids longer than wide; mature proglottids 2–31 (10±5; n = 50) in number, including 0–6 (1.8±1.8; n = 50) proglottids in which testes have atrophied and vas deferens are filled with sperm. No gravid proglottids observed on strobila.

Terminal proglottid (Fig. 10C): 250–650 (422±84; n = 49) long, 75–215 (141±31; n = 50) wide, length-to-width ratio 1.5–5.9 (3.1±0.9; n = 49). Genital pores marginal, irregularly alternating, 69–86% (77±5; n = 49) of proglottid length from posterior end. Testes in mature proglottids irregularly oval in dorsal view (Fig. 10B), 40–82 (58±9; n = 49; *n* = 75) long, 27–70 (44±8; n = 49; *n* = 75) wide, all in primary field, usually 2, rarely 3 (2.0±0.2; n = 28; *n* = 46) in total number; 1 layer deep, extending from near anterior margin of proglottid to near anterior margin of ovary. Poral testis anterior to aporal testis in proglottids with 2 testes, abutting one another (Fig. 10B). Vas deferens in terminal proglottids somewhat coiled, spanning from near anterior margin of proglottid posteriorly to near ovarian isthmus, entering cirrus sac at medial margin. Cirrus sac elongate oval, bent posteriorly, extending medially to, or near to, midline of proglottid, extending posteriorly to or slightly overlapping anterior margin of ovary, containing coiled cirrus. Cirrus sac in terminal proglottids 50–100 (71±11; n = 49) wide, 35–65 (47±8; n = 49) long. Everted cirrus (Figs. 11J–11L) without conspicuous base, 90–170 (134±30; n = 5; *n* = 6) long, 14–20 (17±3; n = 6; *n* = 7) wide, covered with capilliform filitriches and coniform spinitriches with ridges; coniform spinitriches of cirrus 2.6–3.1 (2.9±0.2; n = 4) long, 0.6–1.1 (0.8±0.2; n = 3; *n* = 5) wide (Fig. 11L). Vagina relatively thick-walled, sinuous, varying in width along its length, with darkly staining cells in walls, extending from ootype along medial line of proglottid to anterior margin of cirrus sac, then laterally to common genital atrium. No vaginal sphincter observed. Proximal portion of vagina slightly expanded. Ovary (Figs. 10B, 10C) near posterior end of proglottid, smooth-margined, usually asymmetrical, H-shaped in frontal view, tetra-lobed in cross-section, maximum width 38–125 (82±20; n = 50); ovarian isthmus located at or anterior to mid-point of ovary. Poral ovarian lobe longer than, equal to, or shorter than aporal ovarian lobe; maximum length of ovary 115–325 (197±52; n = 50). Ovary occupying 33–63% (47±6; n = 49) of proglottid length. Anterior margin of ovary 30–100 (58±17; n = 50) short of genital pore. Mehlis' gland 25–60 (40±9; n = 34) long, 15–35 (25±5; n = 34) wide. Vitellarium follicular; vitelline follicles relatively large, 12–35 (23±5; n = 49; *n* = 84) long, 6–27 (16±4; n = 49; *n* = 84) wide, in 1 dorsal and 1 ventral column on each side of proglottid, extending from posterior margin of proglottid to near anterior margin of proglottid, interrupted ventrally by cirrus sac and vagina. Uterus ventral, sacciform, extending from posterior margin of proglottid to near anterior margin of proglottid.

Free gravid proglottids: Gravid proglottids 560–700 (613±62; n = 4) long, 170–200 (184±14; n = 4) wide. Eggs spherical or semi-spherical. Proglottids not observed en copula.

### Taxonomic summary

#### Type host

*Potamotrygon orbignyi* (Castelnau, 1855) Smooth back river stingray.

#### Additional hosts

*Potamotrygon* sp. (mar1).

#### Type locality

Marajó Bay/Amazon River, Amazon Basin, at Colares, Pará State, Brazil (Lat: 0°55′48″S Long: 48°30′0″W).

#### Additional localities

Cachoeira do Arari, Ilha de Marajó, Amazon basin, at Igarapé Cururu, Pará State, Brazil (Lat: 1°0′36″S Long: 48°57′36″W); Tapajós River, Tapajós sub-basin, Amazon Basin, at Santarém, Pará State, Brazil (Lat: 2°16′48″S Long: 55°0′0″W); Tocantins River, Amazon basin, at Cameta, Pará State, Brazil (Lat: 2°15′0″S Long: 49°29′24″W).

#### Site of infection

Spiral intestine.

#### Holotype

MZUSP No. 6309c (1 whole mount).

#### Paratypes

Forty-nine whole mounts (MZUSP 6302, 6303a–b, 6304a–b, 6305a–h, 6305t, 6306a–b, 6308a–c, 6309a–b, 6309d–h, 6310a–b, 6310f–g, 6311a–c; LRP 7671–7673, 7675–7679; USNPC 104726, 104727 (2 slides), 104729, 104730 (2 slides), 104731–104732), four free gravid proglottids (MZUSP 6307a; LRP 7674; USNPC 104728), 12 scoleces (MZUSP 6282–6293) and 16 proglottids prepared for SEM, cross sections of strobilae of four worms (MZUSP 6924a–g, 6925a–c, 6927a–c, 6929a–d), longitudinal sections of two scoleces (MZUSP 6926a–c, 6928a–c), and seven voucher specimens (i.e., hologenophores) of sequenced worms (MZUSP 6301, 6659–6663, 6961, for GenBank Nos. JF803725, JF803729–JF803734).

#### Vouchers deposited

One-hundred forty-nine whole mounts (MZUSP 6300, 6312–6317, 6319–6321, 6324, 6303c–r, 6304e–q, 6305m–z, 6305za–zf, 6306c, 6307d–g, 6308f–v, 6309m–z, 6309za–zj, 6310e–s, 6311f–m, 6318a–b, 6322a–e, 6323a–b, 6325a–c, 6326a–f, 6327a–e).

#### Etymology

This species is named in honor of the late United States Senator J. William Fulbright, and in recognition of the Fulbright Program he founded to foster mutual understanding among nations through education and cultural exchange. This program helped support collaboration between the authors.

### Remarks

*Rhinebothrium fulbrighti* n. sp. is unique among the three other known species of *Rhinebothrium* from South American potamotrygonids considered valid here in its possession of only 2 (or rarely 3) testes (vs. 4–9 in *R. paratrygoni*, 4–12 in *R. copianullum*, 7–13 in *R. brooksi*). In addition, the possession of 2 (or rarely 3) testes distinguishes *R. fulbrighti* n. sp. from all but 5 of the 38 other species of *Rhinebothrium* considered valid by Healy [54]. It is interesting that the in the five described species that also possess two testes (i.e., *R. biorchidium*, *R. ditesticulum*, *R. rhinobati*, *R. spinicephalum*, and *R. tetralobatum*), all parasitize rays in coastal waters of South America, North America, or the Caribbean Sea. *Rhinebothrium fulbrighti* n. sp. can be distinguished from all of these except *R. rhinobati* in its possession of 2, rather than 1, posteriormost bothridial loculi. *Rhinebothrium fulbrighti* n. sp. has a greater number of proglottids (40–168 vs. 18–33) and loculi (43–53 vs. 22) than *R. rhinobati*. *Rhinebothrium fulbrighti* n. sp. can be readily distinguished from the 2 species of *Rhinebothrium* for which testes data are lacking (i.e., *Rhinebothrium ceylonicum* Shipley & Hornell, 1906 and *Rhinebothrium maccallumi* Linton, 1924), as follows. It is shorter (3.1–18 mm vs. ∼5.8 cm) and has a narrower scolex (320–1110 vs. 4 mm) than *R. ceylonicum*; it is shorter (3.1–18 vs. 28 mm) and possesses more bothridial loculi (43–53 vs. ∼31) than *R. maccallumi*.

The seven specimens of *R. fulbrighti* n. sp. that were included in the molecular analysis were found to nest in a single clade (Clade B, Fig. 2); these specimens exhibited relatively low nucleotide diversity (π = 0.01134) and, hence narrow uncorrected pairwise patristic distance variation (ranging from 0 to 0.02247) in comparison to clades C and D, except *R. paratrygoni* (Clade A, Fig. 2). Our morphological results suggest that *R. fulbrighti* n. sp. is restricted to the lower Amazon, Tocantins and Tapajós rivers. However we were only able to survey haplotypes from the lower Amazon at Marajó Island, so we cannot evaluate whether the genetic cohesiveness we report here is a sampling artifact. Nonetheless, the distinct morphology of this species is reflected by the support we found for this clade. Thus, both datasets support *R. fulbrighti* n. sp. as a distinct evolutionary lineage of freshwater rhibebothriid.

Key to the species of *Rhinebothrium* in Neotropical freshwater stingrays

1a. 2–3 testes per proglottid, *R. fulbrighti*

1b. ≥4 testes per proglottid

2a. Microtriches on cirrus <5 µm in length, *R. paratrygoni*

2b. Microtriches on cirrus >7 µm in length

3a. Anteriormost proglottid as long as wide within anterior third of strobila, *R. brooksi*

3b. Anteriormost proglottid as long as wide within posterior half of strobila, *R. copianullum*

Order RHINEBOTHRIIDEA

*Rhinebothrium* Linton, 1890 (Amended diagnosis)

Euzet [76] provided the most recent diagnosis of the genus *Rhinebothrium*, noting a lack of morphological consistency among its many species, a concern also stated by Healy [53]. The four species of cestodes described or redescribed here are rhinebothriines, based on their possession of stalked bothridia, and are generally consistent with the generic diagnosis for *Rhinebothrium* of Euzet [76]. However, this designation requires modification to accommodate the features of the bothridia seen in *R. copianullum* and *R. brooksi*. The following revised diagnosis of *Rhinebothrium* is proposed (differences from the diagnosis of Euzet [76] are indicated in bold):

Rhinebothriidea: Scolex with four pedunculated bothridia, subdivided into loculi by several transverse septa, and by one medial, **or by one medial and two lateral**, longitudinal septa. Bothridial margin entire or loculated. Myzorhynchus absent. Cephalic peduncle short or absent. Strobila acrapedote or craspedote. Euapolytic. Genital pore lateral. Testes few to numerous, anterior to cirrus sac; postvaginal testes absent on poral side. Ovary posterior, **H-** or X-shaped in cross-section. Vagina anterior to cirrus sac. Vitelline follicles lateral. Uterus simple, median. Eggs isolated or in cocoon. In batoids. Cosmopolitan.

#### Type species

*R. flexile* Linton, 1890.

#### Additional species

*R. abaiensis* Healy, 2006, *R. baeri* Euzet, 1959, *R. biorchidum* Huber & Schmidt, 1985, *R. brooksi*, *R. burgeri* Baer, 1948, *R. cadenati* Euzet, 1954, *R. ceylonicum* Shipley & Hornell, 1906, *R. chilensis* Euzet & Carvajal, 1973, *R. chollaensis* Friggens & Duszynski, 2005, *R. copianullum* Reyda, 2008, *R. corymbum* Campbell, 1975, *R. devaneyi* Brooks & Deardorff, 1988, *R. ditesticulum* Appy & Daily, 1977, *R. euzeti* Williams, 1958, *R. fulbrighti*, *R. ghardaguensis* Ramadan, 1984, *R. gravidum* Friggens & Duszynski, 2005, *R. hawaiiensis* Cornford, 1974, *R. himanturi* Williams, 1964, *R. hui* (Tseng, 1933), *R. kinabatanganensis* Healy, 2006, *R. leblei* Euzet & Carvajal, 1973, *R. lintoni* Campbell, 1970, *R. maccallumi* Linton, 1924, *R. margaritense* Mayes & Brooks, 1981, *R. megacanthophallus* Healy, 2006, *R. monodi* Euzet, 1954, *R. oligotesticularis* (Subramaniam, 1940) Healy, 2006, *R. paratrygoni* Rego & Dias, 1976, *R. pearsoni* Butler, 1987, *R. rhinobati* Daily & Carvajal, 1976, *R. scobinae* Euzet & Carvajal, 1973, *R. setiensis* Euzet, 1955, *R. spinicephalum* Campbell, 1970, *R. taeniuri* Ramadan, 1984, *R. tetralobatum* Brooks, 1977, *R. tumidulum* (Rudolphi, 1819), *R. urobatidium* (Young, 1955) Appy & Dailey, 1977, *R. verticillatum* (Subhaprada, 1955) Ramadan, 1984, *R. walga* (Shipley & Hornell, 1906) Euzet, 1959, *R. xiamenensis* Yanhai & Wenchuan, 2001.

## Discussion

### Species delimitations and patterns of intra-specific morphological variability

Our criteria to delimit species within freshwater lineages of *Rhinebothrium* were based on phylogenetic patterns of monophyly recovered from nucleotide data of a single locus associated with morphological cohesion. Although we acknowledge that species could be recognized in the absence of reciprocal monophyly [77], [78], to address the problems frequently associated with lineage sorting –, which prevent us to equate gene trees to species trees – would require data that is not only unavailable at this moment but also not trivial to obtain (e.g., mutiple luci data, appropriated sample design, generation time information, among others, see [17] and references therein). Nonetheless, we think that we provided a valuable contribution to our understanding of the diversity of freshwater lineages of *Rhinebothrium*.

The morphological data and molecular phylogenetic hypothesis for a single locus together provided the evidence we have to recognize four lineages, or putative species, of the cestode genus *Rhinebothrium* in the many freshwater stingray species we examined throughout the Amazon and La Plata basins (see Table S1 and Fig. 1). The specimens we examined had a greater amount of intra-specific variation for certain morphological characters (e.g., in total length, number of proglottids), and a lower host specificity, than is typically documented in cestodes from marine elasmobranchs (see below), raising the possible objection that we have failed to recognize cryptic species. We would argue, however, that each of the four species is a distinct and recognizable unit based on unique combinations of morphological features and that comprized a clade of COI sequence data. Note that the largest variation in total length documented on our phylogenetic hypothesis resides in Clade A – *R. paratrygoni*, in which the worms ranged from 9 to 80 mm in length. However, this clade exhibited the lowest nucleotide diversity and the narrowest range of pairwise distances (0.00778 and 0–0.01889, respectively). Thus, although the haplotypes of Clade A seem to possess high molecular cohesiveness, they vary greatly in length and number of proglottids. On the other hand, the second largest nucleotide diversity and the widest range of pairwise distances (0.06796 and 0–0.14442, respectively) was observed in Clade D, assigned to *R. copianullum*. Yet, in terms of total length, these worms seem to display less variation (13 to 37 mm among sequenced specimens that were measured) than what was observed for *R. paratrygoni*. Thus there appears to be no correlation between molecular and morphological variation within species of *Rhinebothrium* in Neotropical freshwater stingrays. We believe that the variation encountered in this study is higher than usually documented because we examined more specimens, from more hosts and localities, than is typical. We do not know if this pattern is confined to parasites of potamotrygonids, but recommend caution on the use of worm length and number of proglottids to diagnose marine species of tetraphyllideans.

Throughout the recent epistemological development of Systematics, many authors have devoted extensive time and effort to discuss theory and methods of phylogenetic inference and the theory of species concepts [79]–[81]. Conversely, the same amount of effort has not been directed to discussion of operational methods for species discovery [3], [82]. Despite the existence of tree-based operational criteria for delimiting species (reviewed in [16]), and the ongoing recent development of methods that incorporate macro and microevolutionary patterns of diversification into robust protocols of species discovery [14], [15], [83]–[87], these methods remain widely unused for certain groups. Species delimitation in tetraphyllideans, for example, has rested traditionally on nontree-based methods. Typically discrete morphological attributes are utilized, but in some cases, recognition of morphological discontinuities are used if no discrete morphological attribute is recognized for a given new taxon. Although, systematists typically favor phylogenetic methods to delimit species [16], we recognize that boundaries of morphological discontinuities still have its place in taxonomy and systematics as long as morphological discontinuities exist and can serve as criteria by which individuals can be tested for species membership. After all, the biological significance of what we define as species relies on the assumption that the taxon to correspond to distinct evolutionary lineages subject to test as new data and methods become available. Be that as it may, our results suggest that the recognition of morphological discontinuities is highly dependent on sample size and/or biogeographical representation.

Additional biological material from which more data could be extracted might reveal in the future that there were hidden lineages to which we can assign the rank of species that we were not able to recognize. That is what systematics is all about, a circle of reciprocal hypotheses testing. Our results suggest that the presumed boundaries based on morphological discontinuities that once were used to justify species within this group seem not to exist in nature. That is, the sizes represented among the mature *R. copianullum* redescribed here, as well as the sizes represented among the specimens sequenced in Clade D (Fig. 2), do not represent multiple species, though it would seem intuitive to recognize them as such. Our results suggest that if species boundaries ought to be defined on the basis of morphological discontinuities, one has to make sure that the biological material available to apply such a criterion represents the intra-specific variation of the lineage. For parasites of potamotrygonids, we find that a good representation of intra-specific variation can only be achieved by meaningful representation of hosts and biogeographical region, in conjunction with careful evaluation of a diversity of characters, including microthrix data.

### *Monophyly* of *Rhinebothrium* Linton, 1890

Although our primary concern in this contribution was to expand the knowledge of freshwater lineages of *Rhinebothrium* that inhabit the potamotrygonids of South America, the phylogenetic pattern recovered from the phylogenetic analyses of COI nucleotide sequence data suggest that this genus as a whole requires revision. As depicted in Fig. 2, *Rhinebothroides* was found to nest within a clade represented by the freshwater *Rhinebothrium* species, although with poor bootstrap support. Similarly, several marine species of *Rhinebothrium* were found to be more closely related to yet other rhinebothriidean taxa (e.g., *Scalithrium*) and thus undermine the monophyly of *Rhinebothrium*. *Rhinebothroides* species are endemic to potamotrygonids, and are morphologically distinguishable from *Rhinebothrium* species in potamotrygonids in that their proglottids have highly asymmetrical ovaries, a posteriorly positioned genital pore, and >20 testes (see [47], [49]). Thus, emendation of the diagnosis of *Rhinebothrium* to accommodate members of *Rhinebothroides*, although potentially necessary, may be premature. It could be argued, for instance, that the evidence for the polyphyly of *Rhinebothrium* presented here is weak since our taxonomic representation is far from adequate to address the problem, and the use of a single locus is known to be a poor estimator of species trees [77], [88]–[90]. Nonetheless, the phylogenetic pattern recovered here is similar to the results of Healy *et al.* [91] based on a broader spectrum of rhinebothriidean taxa and sequence data for two other loci. Healy *et al.*'s [91] analyses were based on ssrRNA and lsrRNA nucleotide data for multiple species from each rhinebothriidean genus. Their results also supported the polyphyletic status of *Rhinebothrium* and the close phylogenetic association between freshwater lineages of *Rhinebothrium* and *Rhinebothroides* species.

Healy [53] emphasized that while *Rhinebothrium* ought to be split into multiple genera, further study is required in order to identify synapomorphies that unite monophyletic subsets of species in the genus. Similarly, we feel that additional molecular and/or morphological data from broader taxonomic representation ought to be compiled and analyzed to explore the circumscription of monophyletic assemblages before any taxonomic actions are formally taken.

### Patterns of biogeographical distribution and host specificity

The geographic sampling for this study was extensive, including 20 rivers (or lakes) in the Amazon Basin, and seven rivers in the La Plata Basin (see Fig. 1). The diversity of stingray species was also extensive, including 14 recognized and 18 potentially undescribed species. The extent of the survey makes it possible to characterize the distribution patterns of each of the four species within these two basins, as is done below. However, sampling of additional basins (e.g., the Orinoco and Magdalena river basins) is needed for a more complete picture of *Rhinebothrium* distribution in South America. The four *Rhinebothrium* species recognized here exhibited different geographic distributions and levels of host specificity. Intriguingly, the distribution patterns are somewhat congruent with patterns of other aquatic organisms in South America.

*Rhinebothrium fulbrighti* appears to be biogeographically restricted to the lower Amazon, despite the more widespread occurrence of its type host *Potamotrygon orbignyi* [24]. *Rhinebothrium fulbrighti* was only encountered in *Potamotrygon orbignyi* and *Potamotrygon* sp. (mar1) from Marajó Island, and from *P. orbignyi* from the lower portions of the Tocantins and Tapajós rivers. It was not encountered in other localities in which the type host was sampled, such as the Rio Negro (see Table S1). Several factors, ranging from historical to ecological, may be responsible for the restricted biogeographic distribution of *R. fulbrighti*, but too little is known about this system at this time to investigate this. For example, no complete life cycle is known from any potamotrygonid cestode to date; not a single intermediate host has been identified in the literature. Like *R. fulbrighti*, the stingray monogenean *Potamotrygonocotyle auriculocotyle* is restricted to the lower Amazon [92], even though one of its host species, *P. motoro*, occurs elsewhere [19]. The distribution reported for these two potamotrygonid parasites could be considered a restricted lowland distribution, similar to the lowland distributions of many other organisms, such as several species of characiform fishes [93].

The distribution of *R. brooksi* also appears to be somewhat restricted with respect to the distribution of its hosts. *Rhinebothrium brooksi* commonly occurs in the Rio Negro, but was also encountered in the Xingú and Tapajós river basins, although rarely. The two host species reported for *R. brooksi*, *P. aiereba* and *P. orbignyi*, occur in several other rivers in the Amazon Basin (e.g., Madre de Dios, Yavari) where *R. brooksi* was not encountered. To our knowledge, no other parasite of potamotrygonids parallels the distribution of *R. brooksi*, but several species are restricted to the Rio Negro, such as *Rhinebothroides moralai* [94], and the monogeneans *Potamotrygonocotyle quadrocotyle* and *Potamotrygonocotyle umbella* [92]. The Rio Negro was historically connected to the Orinoco River Basin [95] and the two rivers together represent an area of endemicity that corresponds to the distribution of several characiform fishes [93]. Because both *P. aiereba* and *P. orbignyi* also occur in the Orinoco River Basin [19], one might expect to find *R. brooksi* in that basin as well, calling for future studies of potamotrygonid parasites in the Orinoco.

*Rhinebothrium copianullum* and *R. paratrygoni* is each more widely distributed and less host specific than both *R. fulbrighti* and *R. brooksi*. We found that *R. copianullum* reaches maturity in *Paratrygon aiereba*, the host species in which most of the *R. copianullum* specimens encountered in this study were found, but mature specimens were also found in seven species of *Potamotrygon*. The geographic distribution of *R. copianullum* includes the lowlands and the Brazilian Shield; within these areas it was encountered in nearly all of the sampled sites in the Amazon Basin (Fig. 1), except for the easternmost PA07 site (Confluence of Poty and Parnaiba rivers) and a few other localities. The widespread distribution of *R. copianullum* exceeds the widespread distribution of *P. aiereba*; its distribution also includes localities in which only endemic ray species were encountered, such as *Potamotrygon* sp. (tpj2) in the Teles Pires River (Tapajós Basin, TO05, see Table S1). Other potamotrygonid parasites that are widely distributed are in fact even more widely distributed than *R. copianullum*, i.e., they are not restricted to a single basin. Four species of the monogenean genus *Potamotrygonocotyle*, and the cestode *Rhinebothroides freitasi* occur in both the Amazon and La Plata basins, while *R. venezuelensis* and *R. glandularis* occur in the Orinoco, Amazon, and La Plata basins [47], [92], [94]. This distribution pattern of these other widely distributed cestodes and monogeneans raises the possibility that *R. copianullum* may occur in more than one basin. The extensive survey data presented here suggest that *R. copianullum* does not occur in the La Plata River Basin, but more collections are needed to address the possible occurrence of *R. copianullum* occurring elsewhere, such as in the Orinoco River Basin.

The six different species of *Potamotrygon* in which *Rhinebothrium paratrygoni* was found to reach maturity consist of four species that occur throughout the La Plata Basin, and two species that occur in the western portion of the Amazon Basin (ACO6, see Fig. 1). This distribution has not, to our knowledge, been observed for other species of potamotrygonid parasites, but the distribution of the cestode *Rhinebothroides venezuelensis* is similar in that it is found in the La Plata Basin, and in the Western Amazon Basin [94], but it also occurs in the Orinoco. Distribution patterns similar to *R. paratrygoni* are known for some fishes and other organisms. In their study of patterns of northern cis-Andean South American freshwater fishes, Lima and Rebeiro [95] provided multiple examples of teleosts with distributions that consist of the La Plata Basin and the western portion of the Amazon Basin, including a species of *Brycon* (Characiformes), species of the siluriform genera *Lepthoplosternum* and *Otocinclus*, and a species of *Pseudotylosurus* (Beloniformes). Other organisms, such as several species of trichodactylid crabs, have similar distributions [95]. These patterns are formally termed foreland basin distributions, corresponding to the elongated, tectonically imposed lowlands situated between the Andes to the west, and the Brazilian Shield to the East [95]. Forelands have historically had constant hydrographic change, either by headwater-capture [96] or by megafan dynamics [97], and have also been subjected to marine incursions [98], potentially resulting in these widespread distribution patterns of organisms. Organismal distributions that span the divides of one or more basins can be interpreted as evidence of historical relationships among foreland basins (see [95]), or simply as evidence of dispersal routes [92].

None of the four species of *Rhinebothrium* examined in detail here appear to exhibit strict, oioxenous host specificity (sensu Euzet & Combes [99]). The number of potamotrygonid species in which each *Rhinebothrium* species was found to reach maturity ranged from two species for *R. fulbrighti*, to eight species in two genera for *R. copianullum*. *Rhinebothrium* species typically parasitized more than one potamotrygonid species at each locality, but at each locality certain potamotrygonid species appeared to be more important as a host resource than others. In Rio Negro, for example, mature specimens of *R. brooksi* were recovered from 20 of the 39 specimens of *P. aiereba* examined, but in only one of the 51 specimens of *Potamotrygon orbignyi* examined. Survey data also suggest that *Rhinebothrium* species may not be able to reach sexual maturity in all of the potamotrygonid species they are able to infect. For example, immature, but no mature, specimens of *R. copianullum* were encountered in *P. motoro*, *P. schroederi* or *Potamotrygon tatianae*, despite the fact that the number of individuals of each of these species sampled in the Amazon Basin ranged from 14 to 101 (see Table S1). In summary, although *Rhinebothrium* species exhibit strict host specificity for potamotrygonids, our survey data suggest that each species has some degree of host preference among potamotrygonid species.

The degrees of host specificity observed in this study differ markedly from the high levels of host specificity represented for marine species of *Rhinebothrium* [54], [100], [101]. Most of the 38 species of *Rhinebothrium* that parasitize marine elasmobranchs inhabit only a single species [53]. In fact, a high level of host specificity has been documented for marine elasmobranch cestodes in general [13], [102]–[104]. For example, the majority of the 201 species of onchobothriid cestodes reviewed by Caira and Jensen [13] exhibited oioxenous specificity for their elamobranch hosts.

Given that many of the reports of oioxenous specificity in elasmobranch cestodes have been generated in the context of survey work (e.g., Borneo, Baja California) involving examination of multiple host species, it seems reasonable that the difference between the lower level of host specificity reported here for *Rhinebothrium* species of potamotrygonids and the higher level of host specificity reported elsewhere for cestodes of marine elasmobranchs is real. If so, both ecological and historical factors unique to this freshwater system are likely to have influenced the pattern we see in this freshwater system. As Poulin [105] stated, host specificity is essentially a form of resource specialization for the parasite. In this freshwater host-parasite system, *Rhinebothrium* species can be viewed as specialists to the degree that the habitat they require to reach sexual maturity is the spiral intestine of specific potamotrygonid species. On the other hand, *Rhinebothrium* species parasitizing potamotrygonids can also be viewed as generalists to the degree that they are able to develop in the spiral intestine of more than one stingray species. From a historical standpoint, low degrees of host specificity could result either from diversification of host lineages without corresponding speciation in their associated parasite lineage(s), from host switching [105]. Or, perhaps low degrees of host specificity could be related to the potentially young age of this system when compared to marine systems—potamotrygonids are hypothesized to have colonized South America during the Miocene [106]. Yet, none of these explanations can be tested without a robust phylogenetic hypothesis for the Potamotrygonidae, which does not yet exist. The latter explanation can be viewed in terms of causal factors that are either ecological or historical [105], and argued with respect to whether these factors are present in this host-parasite system. For host switching to be successful, novel hosts must be both available and accessible to the parasite via its dispersal mechanisms, and the parasite must be able to establish within the novel host [107]. There is good evidence that multiple potamotrygonid species occur sympatrically [19] and our survey data support this; the localities surveyed each had multiple stingray species present (Table S1). Thus it seems that multiple potamotrygonids species are routinely available to each *Rhinebothrium* species. Potamotrygonid species are also thought to have co-occurred historically in South American rivers. Studies on the geological history of South American rivers have demonstrated that many of the modern rivers have historically shared waters with each other, either as massive lakes [108], or by phenomena such as megafans [97]. Considering the sympatric distributions of potamotrygonid species, it seems likely that novel potamotrygonid host species have been available to enable host switching for *Rhinebothrium* species. It is difficult to pinpoint what factors are involved in making novel potamotrygonid host species accessible via dispersal mechanisms without any information on *Rhinebothrium* life cycles. However, because cestodes are passed to their definitive hosts trophically, it can be inferred that host switching has been facilitated by the overlapping diet of different potamotrygonids species, a pattern reported among potamotrygonids in the Rio Negro [109]. Factors that may have facilitated establishment of *Rhinebothrium* in novel potamotrygonid species could include similarity in physiology among potamotrygonid spiral intestines, but again, this cannot be tested without formal comparison of the physiology of the spiral intestines across potamotrygonids, which has not yet been done. Other factors which may have led to lower degrees of host specificity could relate to variability in survival of host populations at a given locality [105].

### Understanding the origin of marine-derived lineages

Many marine animal lineages besides potamotrygonids have successfully colonized and diversified within the rivers of South America. The descendents of these lineages are major elements of the modern day Neotropical freshwater fauna, and include several lineages of invertebrates [110]–[112], iniid dolphins [113], and a diversity of fishes, such as anchovies, herrings, needlefishes, flatfishes, drums, as well as the potamotrygonid stingrays [98]. These marine-derived lineages have been the focus of studies in which possible marine origins and mechanisms of colonization have been investigated (e.g., [106], [114]–[117]). Both dispersal and vicariance have been invoked as possible colonization mechanisms of such fauna. In the case of the potamotrygonids, novel studies (e.g., Brooks *et al* [35]) in which parasite phylogenies were used to infer host phylogenies, sparked much discussion, and resulted in the development of an intriguing set of hypotheses. In one scenario, rays were thought to have colonized a Pacific-draining Proto-Amazon between the early Cretaceous and Mid-Miocene [35], [117]. In another scenario, which has been supported by studies of other marine derived lineages (see review by Lovejoy *et al* [98]), rays were hypothesized to have moved from the Caribbean to the upper Amazon via marine incursions during the Miocene [28], [106]. Relationships among cestodes were used to explore both scenarios.

As our study shows, more work is needed to build a robust phylogenetic hypothesis for the cestodes before the origin of this intriguing group can be investigated. The relationships between the four *Rhinebothrium* species detailed here, species of *Rhinebothroides*, and their marine relatives need to be further investigated by implementing broader taxon sampling, additional loci, and additional morphological data. Until this can be done, the identity of a potential marine sister taxon to this group of cestodes will remain unclear. In addition, more study is needed in other basins, such as the Orinoco and Magdalena, before historical and ecological factors can be proposed to explain what seem to be complex biogeographic patterns of species distributions. Although we can offer no potential explanation of how (or where) the ancestors of this fascinating group colonized South America, our study shows that the colonization of freshwater was followed by extensive diversification, and that the resulting lineages are well-established components of South American rivers.

## Supporting Information

Table S1 **Potamotrygonid stingray species and localities sampled in this study.**
(XLS)

Table S2 **Cestodes sequenced, with taxon names, hosts, collection localities, and museum voucher numbers for hologenophores.**
(XLS)

Table S3 **Maximum likelihood, AICc, and BIC scores for 5 sets of substitution models (subsets) and 3 partition schemes based on codon position for COI. Gray lines correspond to the lowest scores achieved by AICc and BIC model selection criteria.**
(XLS)
